# Mechanism of subsidence of the Northeast Japan forearc during the late period of a gigantic earthquake cycle

**DOI:** 10.1038/s41598-019-42169-y

**Published:** 2019-04-05

**Authors:** Ryohei Sasajima, Bunichiro Shibazaki, Hikaru Iwamori, Takuya Nishimura, Yoshihiko Nakai

**Affiliations:** 1 International Institute of Seismology and Earthquake Engineering, Building Research Institute, Tsukuba, 305-0802 Japan; 2 Disaster Prevention Research Institute, Kyoto University, Uji, 611-0011 Japan; 3 Earthquake Research Institute, The University of Tokyo, Tokyo, 113-0032 Japan; 4 Department of Solid Earth Geochemistry, Japan Agency for Marine-Earth Science and Technology, Yokosuka, 237-0061 Japan; 5 Department of Earth and Planetary Sciences, Tokyo Institute of Technology, Tokyo, 152-8551 Japan

## Abstract

The forearc in Northeast Japan subsided (3–4 mm/year) in the interseismic ~100 years before the 2011 Tohoku earthquake (M_W_9.1) just like it did during this event. This study attempts to understand the mechanism of the vertical displacement of the forearc during gigantic earthquake cycles via numerical modeling. The results suggest that the interseismic subsidence rate in the forearc increases with the duration of the locking of the asperity of the gigantic earthquake over several hundred years, due to the increasing slip deficit rate on the deeper parts of the plate interface. The increasing slip deficit rate is caused by both the decreasing the shear stress in the shear zone owing to the continuous locking of the asperity and the increasing the mobility of the continental lithosphere owing to the viscoelastic relaxation in the mantle wedge. The deep slip deficit rate extending to ~100 km depth of the plate interface is necessary to explain the observed interseismic forearc subsidence rate. The results also suggest hundreds of years of continuous locking of the asperities of a gigantic earthquake in the western Kuril subduction zone, where fast forearc subsidence has been observed as well.

## Introduction

The Northeast Japan (NEJP) arc is an island arc located around the subduction zone where the old Pacific plate subducts from the Japan Trench at a rate of 8.3–8.4 cm/year^1^. This subduction zone recently experienced a gigantic earthquake, called the 2011 Tohoku earthquake (M_w_9.1), in which a very large fault slip (50–65 m) occurred on the shallow plate interface near the trench (e.g., refs^2,3^). Similar past events with recurrence interval of ~600 years were identified based on the tsunami deposits^4^.

**Figure 1:**
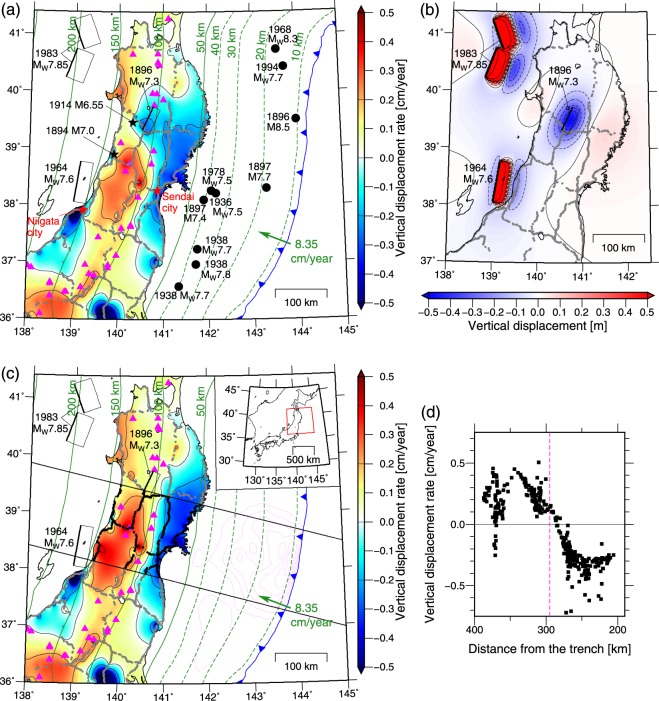
(**a**) Vertical displacement rate (${\dot{U}}_{z}$) in the NEJP island arc obtained through leveling measurements^6^. The observation period ranged from 1892–1906 to 1986–1999 for the NEJP region^6^. The 0.1 cm/year of the absolute uplift rate of the origin of the leveling^14^ was corrected. The small gray dots indicate the benchmarks (observation points) of the leveling. The colors and black contours indicate the interpolated ${\dot{U}}_{z}$ by leveling adapted from Nishimura^5,55^ using the interpolation method by Smith & Wessel^56^ and implemented in the Generic Mapping Tools (GMT, http://gmt.soest.hawaii.edu/)^57^. The contour interval is 0.2 cm/year. Green solid contours and dashed contours indicate the depth of the Pacific slab’s upper surface^58–60^. Black circles indicate the epicenters of major earthquakes at the plate interface during the observation period^19,30,61^. Rectangles and black stars indicate the fault planes and epicenter, respectively, of the major inland earthquakes^16–18,30,62^ in the NEJP region. Pink triangles mark the active volcanoes by Japan Meteorological Agency^63^. Blue line indicates the Japan Trench. Green arrow indicates the plate convergence rate and direction^1^. (**b**) Modeled co- and post-seismic vertical displacement of the three major inland earthquakes during the observation period. The contour interval is 0.1 m (shown at $\le |0.5|$ m) and 1 m for the dotted and solid lines, respectively. (**c**) ${\dot{U}}_{z}$ by the leveling that the modeled co- and post-seismic vertical displacement of the three inland earthquakes shown in (**b**) was subtracted. Data used in this study are indicated using black dots in the range marked by the two black lines. Pink contours indicate the coseismic slip of the 2011 Tohoku earthquake^2^ at 10 m intervals. (**d**) The used leveling data as a function of the distance from the trench. Black squares indicate the ${\dot{U}}_{z}$ based on leveling that are indicated by black dots in (**c**). Pink dashed line marks the volcanic front. The typical 1σ uncertainty of the observed ${\dot{U}}_{z}$ is 0.03–0.06 cm/year (see text in the *Leveling data* section). Figure was created using the GMT^57^.

The vertical displacement rate on the middle NEJP island region (38–39.5°N) above the deeper extended portion of the large coseismic slip area of the 2011 Tohoku earthquake is characterized by fast subsidence (3–4 mm/year) in the forearc and uplift in the backarc during the interseismic period^5^ (Fig. 1). These features were observed by both leveling measurements during 1892–1999^5,6^ (Fig. 1) and the dense Global Navigation Satellite System (GNSS) network during 1996–2011^5^. The forearc region subsided by up to 1.2 m at the 2011 Tohoku earthquake^5^, and it has been rapidly uplifted during the postseismic period^5^. The mechanism of the vertical displacement of the NEJP forearc during gigantic earthquake cycles has been argued and is still moot^5,7–9^.

Several studies have attempted to explain the observed interseismic subsidence rate in the forearc region via kinematic modeling of the crustal deformation caused by interplate coupling and earthquake cycles. The models assuming an elastic half-space suggest that very deep interplate coupling, which is 50–30% of the coupling ratios at 50–100 km depth, is necessary to produce the observed interseismic subsidence rate in the forearc^5,10^. The coupling ratio is defined as $\frac{{v}_{pl}-v}{{v}_{pl}}$, where *v*_*pl*_ and *v* indicate the long-term plate convergence rate and interseismic fault slip rate, respectively. The models assuming a layered elastic and viscoelastic structure suggest that only shallow (<40 km depth^8^ or <25 km depth^9^) interplate coupling can produce the observed interseismic subsidence rate^8,9^. Therefore, different assumptions of the rheological structure yield substantially different results for the deep interplate coupling to explain the observed forearc subsidence rate. Therefore, using realistic rheological structures for modeling is needed to reveal the mechanism of the interseismic forearc subsidence.

Locking of asperities on the plate interface causes a slip deficit rate $({v}_{sd}={v}_{pl}-v)$ not only on the asperities, which has 100% of the coupling ratio, but also on the surrounding frictionally stable portion, where fault creeps and the coupling ratio is between 0% and 100% (e.g., ref.^11^). Therefore, the estimated partial coupling ratio on a deeper part of the plate interface in previous studies^5,10^ suggests slip deficit caused by locking of the asperities at the shallower parts. Deeper in the plate interface, the behavior shifts from frictional slip on the fault plane to volumetric and ductile deformation in shear zones, with increasing temperature at the plate interface. This is based on the geological evidences of the fossil shear zone^12^. The ductile deformation rate in the shear zone may also contribute to the interseismic deformation of the NEJP forearc above the deep part (50–100 km depth) of the plate interface. Therefore, considering the segmentation of asperities, frictionally stable parts (fault creep parts), and ductile shear zones is important to understand the mechanism of the vertical displacement in the forearc during gigantic earthquake cycles.

This study aims to analyze the physical mechanisms beyond the vertical displacement in the NEJP forearc during gigantic earthquake cycles. Clarifying it will deepen understanding of the deep interplate coupling and afterslip mechanisms in gigantic earthquake cycles. Furthermore, it is important to understand these phenomena for estimating the potential of future gigantic earthquakes in the western Kuril subduction zone, where fast interseismic forearc subsidence has been observed as well^10^ and the last gigantic earthquake occurred in the 17th century^13^.

**Figure 2:**
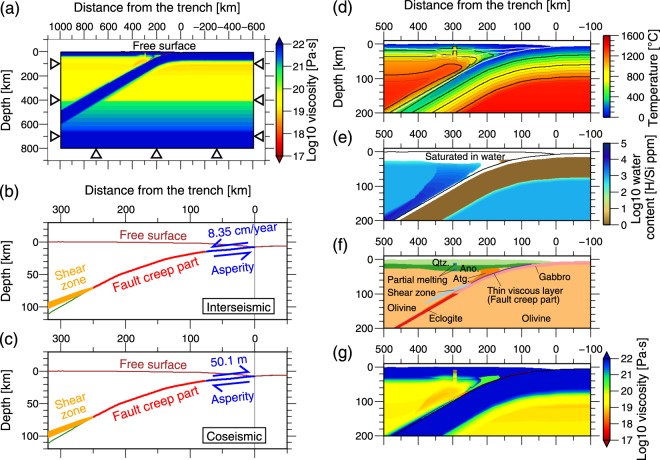
Boundary conditions and rheological structure of the finite-element model. (**a**) The finite-element model with the viscosity and boundary conditions. Triangle indicates a fixed boundary condition. (**b**) Boundary conditions on the plate interface for interseismic period. Blue arrows indicate the dislocation for the backslip of full locking applied using the split-node method^38^. Fault creep part is composed of a thin (~3 km) viscoelastic layer with low viscosity and creeps almost freely during the interseismic period. Green line indicates the slab’s upper surface. (**c**) Coseismic boundary condition on the plate interface. Blue arrows indicate the dislocation for the earthquake slip applied using the split-node method^38^. (**d**) Thermal structure for the rheological structure. Contour interval is 100 °C and 400 °C for the thin and thick lines, respectively. White lines mark the Conrad, Moho, and plate interface. (**e**) Water content in olivine for the rheological structure. We assumed that minerals in the continental crust were saturated in water and antigorite and minerals in the oceanic crust were under wet conditions (white areas). Black lines mark upper surface of the continental lithosphere and plate interface. (**f**) Lithological units for the rheological structure. Qtz.: Quartz, Ano.: Anorthite, Atg.: Antigorite. (**g**) Rheological structure (viscosity distribution) used in the modeling based on (**d**–**f**), pressure, water-fugacity, assumed strain rate, and the flow laws of minerals. (**b**–**g**) show the enlarged view focusing on the subduction plate boundary. Figure was created using the GMT^57^.

For this purpose, we built a model of the crustal deformation due to interplate coupling and earthquake slip during earthquake cycles using the finite-element method. The model considers a realistic heterogeneous rheological structure, the frictionally stable part (fault creep part) at the plate interface via a thin low-viscosity layer, and a deep viscoelastic shear zone (Fig. 2).

## Leveling Data

This study attempted to explain the interseismic vertical displacement rate (${\dot{U}}_{z}$) in the middle NEJP island arc (38–39.5°N) during ~100 years before the 2011 Tohoku earthquake observed by leveling. To focus on the crustal deformation that is associated with the gigantic earthquake cycles of the 2011 Tohoku earthquake (M_W_9.1), we define the range of leveling data used in this study by two lines that are parallel to the arc-perpendicular direction at the center of the range so that the range corresponds to the surface projection of the large coseismic slip area (>30 m) of the 2011 Tohoku earthquake^2^ (Fig. 1(c)).

We used the leveling data analyzed by Kunimi *et al*.^6^. ${\dot{U}}_{z}$ was obtained by taking the difference between two leveling observations and dividing the difference by the time period between the two observations, assuming that ${\dot{U}}_{z}$ is constant with respect to time. This is a reasonable assumption for this study (Supplementary Fig. S1). One leveling survey required up to 15 years to reach completion^6^. The earliest and the latest leveling surveys whose data were analyzed by Kunimi *et al*.^6^ in the NEJP region were conducted during 1892–1906 and 1986–1999, respectively^6^. We assumed that the time between the two observations is the difference in the middle time of each observation, i.e., 1993 − 1899.5 = 93.5 years. Kunimi *et al*.^6^ reported that the standard deviations of the observed altitudes estimated from the network adjustment of the earliest and latest observations in the NEJP region are 30–50 mm and 10–20 mm, respectively. Therefore, the standard deviation of the estimated ${\dot{U}}_{z}$ is 0.3–0.6 mm/year, assuming linear propagation of the observational error. The origin (reference point) of the leveling data was the Japanese datum of leveling in Tokyo, and was corrected for the vertical deformation caused by the 1923 Taisho Kanto earthquake (M7.9)^6^. In addition, Murakami & Ozawa^14^ indicated that the origin has been uplifting by ~1 mm/year, presumably because of the interplate coupling of the Sagami Trough subduction zone—which includes the source region of the 1923 Taisho Kanto earthquake—estimated from the GNSS and the tidal observations near the origin. Therefore, we added the 1 mm/year uplift rate to all leveling data (Fig. 1(a,c,d) show the corrected ${\dot{U}}_{z}$). The anomalous local subsidence in Sakata City (near 139.8°E, 38.9°N), Niigata City (near 139.0°E, 37.9°N), and the North Kanto district (near 139.7°E, 36.1°N) was caused by groundwater pumping^6,15^ and that in Iwaki City (near 140.8°E, 37.0°N) was caused by coal mining^5^.

The leveling data include co- and post-seismic deformation of large inland earthquakes, including the 1896 Riku-u earthquake (M_W_7.3)^16^ (postseismic only), the 1964 Niigata earthquake (M_W_7.6)^17^, and the 1983 Japan Sea earthquake (M_W_7.85)^18^. We calculated the vertical displacement due to co- and post-seismic deformation of these earthquakes in accordance with Thatcher *et al*.^16^ and subtracted it from the leveling data (Fig. 1). The details are presented in Supplementary Information. We did not subtract the deformation caused by ~M_W_7–8 interplate earthquakes because majority of their co- and post-seismic deformations may be cancelled by their interseismic deformation due to the locking of their asperities because the recurrence interval of majority of the ~M_W_7–8 interplate earthquakes in the study region is shorter than the observation period^19^.

To compare the leveling data to the two-dimensional modeling results, we projected the leveling data in the defined range as a function of the distance from the trench (Fig. 1(d)). The peak of forearc subsidence is located 230–250 km from the trench. This feature was also confirmed by the leveling measurements conducted during 1980–2003 along the other route that is toward the tip of the Oshika Peninsula (near 141.5°E, 38.3°N) and is located near the middle of the defined range^5^. The local subsidence at 370–373 km from the trench corresponds to the subsidence caused by groundwater pumping in Sakata City.

## Results

We study the two-dimensional crustal deformation using the finite element method based on the backslip model^20^, which only models the crustal deformation due to the fault slip that deviates from the long-term plate convergence rate (see *Method*s section). Our model considers only the main asperity of the 2011 Tohoku earthquake located within 75 km of the trench, and the other part of the plate interface mechanically creeps during the interseismic period represented by a thin low-viscous layer^21^ (Fig. 2).

### Model of interplate coupling

**Figure 3:**
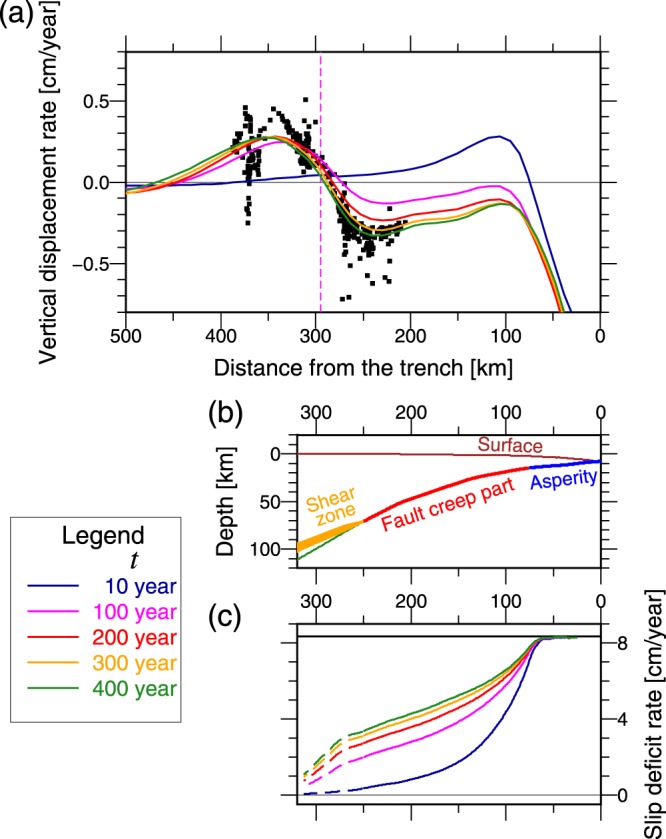
Results of the model of interplate coupling. (**a**) Modeled vertical displacement rate (${\dot{U}}_{z}$) on the surface and observed ${\dot{U}}_{z}$. Colored lines indicate the modeled ${\dot{U}}_{z}$ at each *t*, where *t* is the time from when the locking of the asperity begins. Black dots indicate the observed ${\dot{U}}_{z}$ for ~100 years before the 2011 Tohoku earthquake based on leveling data (same as Fig. 1(d)). Pink vertical dashed line indicates the location of the volcanic front. (**b**) Cross section of the plate interface of the model with boundary conditions. Green line indicates the slab’s upper surface. (**c**) Modeled slip deficit rate (*v*_*sd*_) on the plate interface (solid lines) and across the shear zone (dashed line) at each *t*. Thick horizontal black line (*v*_*sd*_ = 8.35 cm/year) indicates the full interplate coupling. Figure was created using the GMT^57^.

To investigate the manner in which the locking of asperities causes the observed interseismic forearc subsidence, we first model only the interplate coupling. The model begins from an initial condition of zero stress and strain at time *t* = 0, and the locking of the asperity begins at *t* = 0 and continues for *t* > 0 (Fig. 2(b)). Figure 3(a) shows the time evolution of the modeled vertical displacement rate (${\dot{U}}_{z}$) on the surface with the observed ${\dot{U}}_{z}$ by leveling. The subsidence rate increases with the duration of the locking of the asperity for a few hundred years. The modeled ${\dot{U}}_{z}$ for 300–400 years after the locking of the asperity is initiated is similar to the observed ${\dot{U}}_{z}$.

Figure 3(c) shows the time evolution of the modeled slip deficit rate ${v}_{sd}={v}_{pl}-v=-{v}_{model}$, where *v*_*model*_ indicates the fault slip rate (plate convergence slip is positive) in the model based on the backslip model. The modeled slip deficit rate on the fault creep part and across the shear zone is measured as the modeled relative motion across the thin viscoelastic layer and the shear zone, respectively. Soon after the locking of the asperity is initiated (*t* = 10 year), the slip deficit rate on the fault creep part exhibits a similar slip deficit rate on an infinite crack in the elastic space due to the locking of a circular asperity; the slip deficit rate decreases with the distance from the asperity by a square-root form^11^. However, the slip deficit rate on the fault creep part increases and deviates from the square-root form with time. In addition, the slip deficit extends to the shear zone with time. Generally, the slip deficit in the deep plate interface causes the surface above to subside (e.g., refs^5,7^). Namely, the increase in the modeled subsidence rate in the forearc with time is caused by the increasing slip deficit rate in the deep plate interface with time.

**Figure 4:**
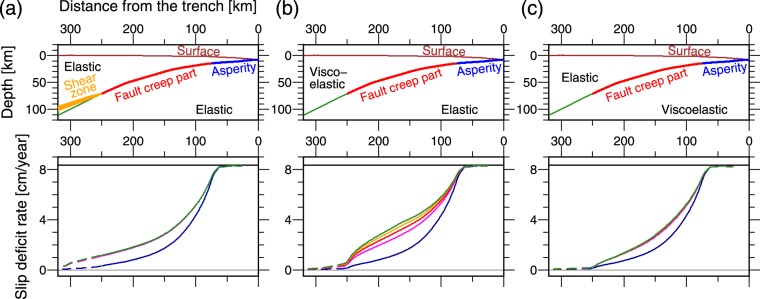
Modeled slip deficit rate based on several test cases of the model of interplate coupling to examine the cause of increasing slip deficit rate with time. Plots are similar to those shown in Fig. 3(b,c) (see the legend in Fig. 3). (**a**) All material is elastic except for the thin viscous layer in the fault creep part and shear zone. (**b**) No shear zone, continental crust and mantle are viscoelastic, which the viscosity distribution is the same as the original model, and oceanic crust and mantle are elastic. (**c**) No shear zone, continental lithosphere and mantle are elastic, and oceanic crust and mantle are viscoelastic, which the viscosity distribution is the same as the original model. Figure was created using the GMT^57^.

To examine the causes of increase in slip deficit rate with time, we model several special cases. The first case is for all model elements to be elastic except for the thin viscoelastic layer in the fault creep part and the shear zone (Fig. 4(a)). The slip deficit rate in the deep part increases during the first 100 years because the slip deficit extends to the shear zone with time (i.e., the locking of the asperity decreases the viscoelastic shear deformation rate in the shear zone compared to the long-term plate convergence rate.). After the slip deficit rate reaches a steady state in an elastic medium (square-root form), no evolution is observed in the slip deficit rate with time. The second case is for viscoelastic continental crust and mantle, which are the same as the original model, elastic oceanic mantle, and elastic shear zone, i.e., no shear zone (Fig. 4(b)). The slip deficit rate in the fault creep part gradually increases and deviates from the square-root form (elastic solution) with time for a few hundred years. This can be explained as follows: the mobility of the continental lithosphere increases as the viscoelastic relaxation in the mantle wedge progresses, i.e., the mantle wedge starts behaving as a viscous fluid. Consequently, dragging the continental lithosphere becomes easier with the duration of the locking of the asperity. The third case is for elastic continental crust and mantle, no shear zone, and a viscoelastic oceanic mantle, which is the same as the original model (Fig. 4(c)). The increment of the slip deficit rate with time is small; therefore, the contribution of viscoelastic relaxation in the oceanic mantle in increasing the slip deficit rate is small.

Therefore, the increase in slip deficit rate with time is mainly caused by (1) decreasing viscoelastic shear deformation rate in the shear zone owing to the shear stress decrement caused by the locking of the asperity and (2) increasing the mobility of the continental lithosphere with progressing viscoelastic relaxation in the mantle wedge. Therefore, the model results indicate that the observed interseismic subsidence in the forearc region was caused by the continuous locking of the shallow asperities located in the large slip area of the 2011 Tohoku earthquake for several hundreds of years with the viscoelastic response in the mantle wedge and shear zone.

We select the optimal viscosity of the shear zone that produces the optimal result to explain the observed ${\dot{U}}_{z}$. The manner in which the changes in viscosity and shape of the shear zone alter the results is presented in Supplementary Information. The results are sensitive to the viscosity of the shear zone (Supplementary Figs S2–S3). None of the models without the shear zone or with the high-viscous shear zone, which have no or very small deep slip deficit rate, reproduces the forearc subsidence even if we change the viscosity of the mantles (Supplementary Figs S4–S7). Therefore, the deep slip deficit rate (*v*_*sd*_/*v*_*pl*_ values of at least 0.35–0.1 for 70–100 km depth) is necessary to explain the observed forearc subsidence rate. Changing the shape of the shear zone does not considerably affect the results (Supplementary Fig. S8). The examinations of changing the other parameters are presented in Supplementary Figs S9–S12.

### Model of earthquake cycles

**Figure 5:**
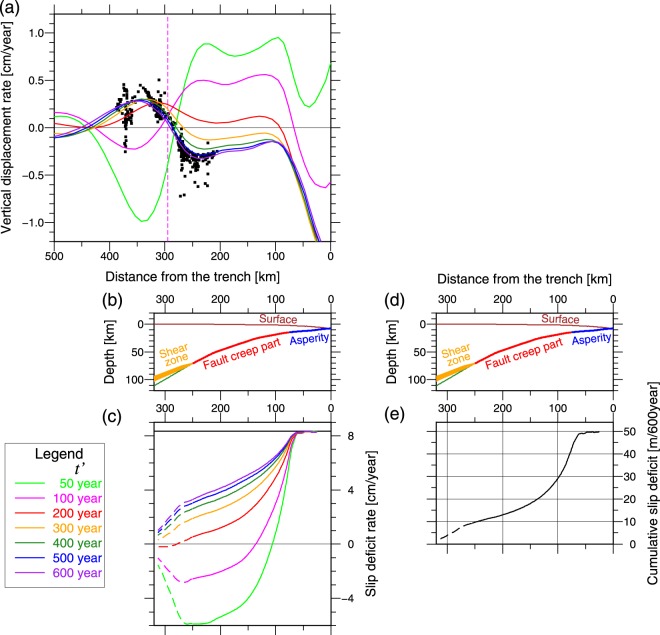
Results of the model of earthquake cycles. (**a**–**c**) The plots are similar to those in Fig. 3(a–c). *t*′ is the time from the fifth earthquake. (**c**) Negative slip deficit rate indicates that the fault slip rate exceeds the long-term plate convergence rate, i.e., it means afterslip. (**d**) The plots are the same to (**b**). (**e**) Modeled cumulative slip deficit during one earthquake cycle. Black curve line indicates the modeled cumulative slip deficit on the plate interface (solid line) and across the shear zone (dashed line) during one earthquake cycle. This was calculated by integrating the positive slip deficit rate during the earthquake cycle. Figure was created using the GMT^57^.

**Figure 6:**
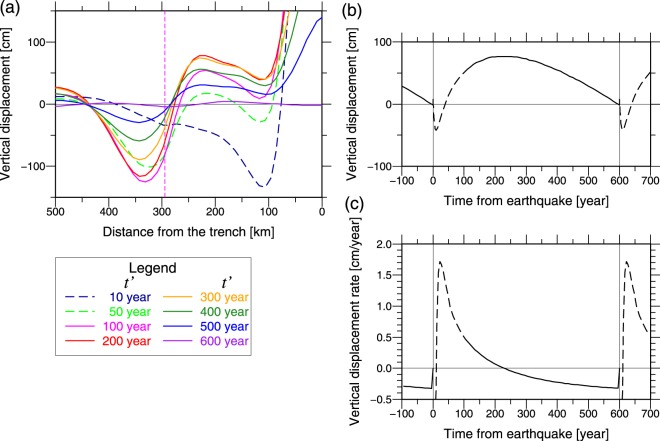
Modeled vertical displacement on the surface in one earthquake cycle. (**a**) Colored lines indicate the modeled cumulative vertical displacement on the surface, which has accumulated since just before the occurrence of the latest (fifth) earthquake at *t*′ = 0. *t*′ is the time from the fifth earthquake. *t*′ = 600 year is the time just before the next earthquake. Vertical pink dashed line indicates the volcanic front. (**b**) Time series of the modeled vertical displacement at the surface near the coastal line (230 km from the trench). Zero of the vertical displacement corresponds to the vertical position just before the latest earthquake occurred at *t*′ = 0. Our model could not represent well the crustal deformation in the earlier postseismic period (dashed line) because there is a delay of several years in the earthquake slip propagation and no transient deformation was considered. (**c**) Time series of the modeled vertical displacement rate at the surface near the coastal line (230 km from the trench). Figure was created using the GMT^57^.

Further, we model the crustal deformation during gigantic earthquake cycles to reveal the mechanism of the vertical displacement during gigantic earthquake cycles. The model’s initial conditions are zero stress and strain at time *t* = 0; further, the earthquake slip occurs every 600 years (Fig. 2(c)), and the first event occurs at *t* = 0. Figures 5 and 6 show the results of the fifth earthquake cycle, which is the cycle that almost reaches a steady state (i.e., the period of the first to fourth earthquake cycles is a spin-up period). Rapid uplift occurs in the forearc region 10–100 years after the earthquake (Figs 5(a), 6(b,c)). Note that several years are required for the earthquake slip to reach the deeper parts of the plate interface because our model represents the fault creep part via a thin viscoelastic layer. Therefore, in the forearc region, subsidence that is caused by the earthquake slip and uplift that is caused by the afterslip and viscoelastic relaxation are mixed during ~10 years after the occurrence of earthquake in our model. This delays the forearc uplift as compared to that observed in a real case in the early postseismic period. Large afterslip and viscoelastic relaxation in the shear zone occur at the deep plate interface for 10–100 years after the earthquake (Fig. 5(c)). The afterslip and the viscoelastic relaxation in the shear zone are mainly responsible for the large uplift of the forearc region 10–100 years after the earthquake. These mechanisms are opposite to the mechanisms of the forearc subsidence in the later interseismic period described in the previous subsection.

The rate of the afterslip exhibits a long decay constant and is controlled by the viscoelastic relaxation rate in the shear zone. Figure 5(e) shows the modeled total slip deficit, which is equivalent to the negative sum of the earthquake slip and total afterslip, in the earthquake cycle. This can be calculated by integrating the positive slip deficit rate during the earthquake cycle. The slip deficit of 10–11.5 m is accumulated during the earthquake cycle in the deeper parts (220–250 km from the trench) of the plate interface in our model. The deeper end of the coseismic slip of the 2011 Tohoku earthquake is located 200–250 km from the trench (~2.5 m coseismic slip at 220 km from the trench) according to the average of the estimated coseismic slip models^3^. Therefore, the maximum total afterslip that is predicted by the model following the 2011 Tohoku earthquake is 9–10 m at 220–250 km from the trench axis. The model in this study predicts that the afterslip with a long decay constant whose rate is controlled by the viscoelastic relaxation rate in the shear zone, continues for ~50 years or more even after the typical afterslip with a short decay constant, which is controlled by the frictional slip law at the plate interface and the cumulative slip follows the logarithm of time^22^, decays. Such long-duration afterslip due to a deep ductile shear zone was suggested by a simulation with rate weakening law at a seismogenic zone, rate strengthening law at a brittle creep zone, and viscous flow at a ductile shear zone^23^. Such long-duration afterslip was also estimated for ~40 years after the Alaska earthquake (M_W_9.2) from geodetic observations^24^.

The afterslip with a long decay constant diminishes, and instead, the slip deficit owing to the locking of the asperity gradually extends to deeper parts ~200 years after the earthquake (Fig. 5(c)). The subsidence rate in the forearc region increases with the slip deficit rate in the deeper parts (Fig. 5(a,c)) because of the continuous locking of the asperity for hundreds of years as described in the previous subsection. The coupling ratios $(\frac{{v}_{sd}}{{v}_{pl}})$ at *t*′ = 600 year, where *t*′ is the time from the fifth earthquake, are 0.5, 0.4, and 0.2 at 45 km, 70 km, and 100 km depths, respectively (Fig. 5(c)). These are similar to the estimated coupling ratios derived from the observed ${\dot{U}}_{z}$, assuming an elastic half-space^5^. Such deep slip deficit is reasonable because deep (up to 100 km in depth) afterslip, which releases the cumulated deep slip deficit, occurs after the 2011 Tohoku earthquake^21,25^.

The cross-sectional images of the modeled velocity vectors in the continental crust and mantle for 100, 250, and 600 years after the earthquake are shown in Supplementary Fig. S13. The modeled horizontal displacement rate in the forearc region at *t*′ = 600 year (3.0–3.7 cm/year) is roughly consistent with the observed interseismic horizontal displacement rate in the forearc region (38–39°N) by the GNSS during 1997–2000 (~3–4 cm/year)^5^. We also made a rough comparison between the modeled vertical displacement rates and the observed ones by the GNSS observations during 1997–2005 in Supplementary Information, and these are roughly consistent with each other (Fig. S14). However, our results cannot strictly be compared to the GNSS data during 1996–2011 because our model does not include the locking of the moderate size asperities (e.g., the ~M_W_7.5 Miyagi-oki earthquake), which did not rupture during 1996–2011, for the middle to deeper part of the plate interface (see *Methods* section and Supplementary Information).

## Discussion

At first, we discuss the limitations of our model. Because the model neglects the locking and earthquake slip of the asperities of the ~M_w_7.5 Miyagi-oki earthquake, our results cannot strictly be compared with short-term geodetic observations whose observation period is shorter than the recurrence interval of the ~M_w_7.5 Miyagi-oki earthquake. Because the model approximately represents the frictionally stable portion via a thin viscous layer, the results cannot explain early periods of afterslip. In addition, we assume a Newtonian viscoelasticity, whereas the actual mineral flow law has power-law stress dependency^26^ especially for the earlier postseismic period (i.e., transient very low viscosity and resulting transient deformation appear just after the earthquake). Therefore, our results underestimate the deformation rate in the early postseismic period and cannot be compared with the observed postseismic deformation for several tens of years after the 2011 Tohoku earthquake. Future numerical modeling that considers fault friction law including the asperities of the ~M_w_7.5 Miyagi-oki earthquake and power-law rheology with modeling absolute stress will resolve this problem. The postseismic transient viscosity recovers to a steady state viscosity within a maximum of 50 years from the occurrence of a large earthquake^27^, i.e., the deformation rate with a Newtonian rheology and that with a power-law rheology become almost identical. Therefore, our model is effective unless we focus on the deformation whose time scales are greater than 50–100 years and that except for the earlier postseismic period. Thus, our model is effective to derive the conclusions and suggestions written in this manuscript and is useless to predict the crustal deformation decades after the 2011 Tohoku earthquake. Because a two-dimensional problem is assumed in this study, our model cannot explain the along-arc variation of the observed vertical displacement rate that decreases with the distance from the range used in this study (>39.5°N and <36°N in Fig. 1(c)). In addition, two-dimensional assumption overestimates the displacement rate for the back-arc, which is far from the source region because of the difference in dimension for geometric decay of the displacement. The backslip model only treats the perturbation of the slip on the plate interface, while the actual deformation rate in the shear zone depends on the absolute stress, which is caused by the steady plate subduction and its perturbation. Therefore, actual time-dependent response of the shear zone may slightly differ from our results.

The model results reveal that a slip deficit at greater depths corresponding to $(\frac{{v}_{sd}}{{v}_{pl}})$ values of 0.5, 0.4, and 0.2 at depths of 45 km, 70 km, and 100 km, respectively (Fig. 5(c)), is necessary to explain the observed vertical displacement rate ~100 years before the 2011 Tohoku earthquake. The total accumulated slip deficit during the gigantic earthquake cycle (Fig. 5(e)) is similar to the coseismic slip distribution of the 2011 Tohoku earthquake^3^ except for the deeper portion (>~50 km depth), where the accumulated slip deficit may have been released by the afterslip following the 2011 Tohoku earthquake.

The model result suggests that the observed fast subsidence of the forearc (3–4 mm/year) occurs only in the later stages of the gigantic earthquake cycle. If the observed forearc subsidence rate had continued through the gigantic earthquake cycle (~600 years), the cumulative interseismic subsidence on the forearc would exceed 2 m. The corresponding cumulative slip deficit in the deeper parts of the plate interface is 24–20 m for 50–70 km depth. In addition, coseismic subsidence of 0.4–1.2 m occurred in the forearc region^5^. Therefore, the postseismic uplift of 2.5–3.5 m due to the afterslip of 20–24 m is required to balance the sum of interseismic and coseismic subsidence. However, the observed postseismic uplift in the forearc region rapidly decays^5^; therefore, the postseismic uplift of 2.5–3.5 m due to the afterslip of 20–24 m is unlikely^5^. Therefore, Nishimura^5^ hypothesized that the observed interseismic fast subsidence rate in the forearc region and the large slip deficit rate in the deeper parts are limited in the later stages of the gigantic earthquake cycle. Our modeling results support this hypothesis and elucidate the physical mechanism.

We predict that the sum of the coseismic subsidence (~1.2 m) and the cumulative postseismic uplift around the coastal line in the forearc region is ~0 m, ~0.5 m, and ~0.75 m at 40 years, 100 years, and 200 years from the 2011 Tohoku earthquake, respectively (Fig. 6(b)). Because there is a delay of several years in the earthquake slip propagation and no transient rheology is considered in our model, the actual time required to recover the coseismic subsidence around the coastal line is probably shorter than 40 years. Similar large postseismic uplift with a long duration, which is ~1.5 m for ~60 years in the east coast of Hokkaido after the gigantic earthquake along the western Kuril Trench in the 17th century, is identified by studying the coastal uplift history^28^. Therefore, a similar large uplift with a long duration will occur after the 2011 Tohoku earthquake (M_W_9.1). Our results suggest that the long-duration postseismic uplift is caused by the long-duration afterslip controlled by the viscoelastic relaxation rate in the shear zone. The long-duration afterslip (up to 9–10 m in total) balances the large slip deficit in the deeper plate interface that accumulates late in the gigantic earthquake cycle (Fig. 5(c,e)), and the resulting long-duration large postseismic uplift in the forearc region balances the large subsidence late in the gigantic earthquake cycle (Fig. 6(b)). This is the proposed model for the vertical displacement in the forearc region during the gigantic earthquake cycle. Our model will be tested by following several hundred years of vertical deformation in the NEJP island arc by geodetic observations. The proposed mechanism can also explain the long-duration large postseismic uplift along the western Kuril Trench in the 17th century.

There is another evidence for the large slip deficit rate in the deeper plate interface in this region in the later stages of the gigantic earthquake cycle. The asperities of the ~M_W_7.5 Miyagi-oki earthquake at 30–50 km depth of the plate interface repeatedly ruptured^19,29^: 1897 M7.4^30^, 1936 M_W_7.5^19^, and 1978 M_W_7.5^19^ (Fig. 1(a)). Yamanaka & Kikuchi^19^ indicated that the slip rate (*v*) on the asperities of the ~M_W_7.5 Miyagi-oki earthquake including its afterslip during 1900–2000 is lower than the half of the plate convergence rate. This is consistent with our result that the modeled slip deficit rate (*v*_*sd*_ = *v*_*pl*_ − *v* for 500–600 years after the gigantic earthquake in this region (30–50 km depth) is 4–5 cm/year (Fig. 5(c)), which is approximately half of the plate convergence rate (*v*_*pl*_). Therefore, we suggest that the large lack of the slip rate on the asperities of the ~M_W_7.5 Miyagi-oki earthquake was caused by the long-term locking of the shallow asperities of the 2011 Tohoku earthquake for several hundred years and was probably compensated by the coseismic slip of the 2011 Tohoku earthquake on the asperities of the ~M_W_7.5 Miyagi-oki earthquake (~8–15 m of the coseismic slip^3^).

The results of this study suggest that the recurrence interval of the ~M_W_7.5 Miyagi-oki earthquake is shorter early in the gigantic earthquake cycle and increases with time because of decreasing loading rate of the asperities of the ~M_W_7.5 Miyagi-oki earthquake with decreasing background slip rate on the deeper plate interface. This will be compared with the recurrence intervals of future Miyagi-oki earthquake. In addition, the predicted large afterslip in this study (up to 9–10 m) will partly be included as the slip rate excess due to the shorter interval of the Miyagi-oki earthquake and its afterslip for the next 50–100 years.

The results suggest that the shear zone is critical to the slip deficit rate and afterslip in the deeper parts of the plate interface during a gigantic earthquake cycle. The modeled vertical displacement rate in the forearc region is very sensitive to the viscosity and width of the shear zone, which control the deformation rate in the shear zone. The results suggest that the absolute deformation rate in the shear zone decreases by few tens percent of the plate convergence rate in the later interseismic period and is much faster than the plate convergence rate in the postseismic period (Fig. 5(c)). The viscoelastic deformation rate in the shear zone is controlled by the magnitude of the shear stress in the shear zone. Therefore, the magnitude of the shear stress in the shear zone substantially varies during the gigantic earthquake cycle; it suggests that the magnitude of the shear stress in the shear zone is unlikely one order of magnitude larger than the stress drop of the gigantic earthquake, which is ~6 MPa for the spatially averaged stress drop of the 2011 Tohoku earthquake^31^. This helps to understand the rheological properties and behavior of the shear zone in the subduction zones.

The range of the observed fast subsidence in the forearc region coincides with the extent of the dip of the large slip area of the 2011 Tohoku earthquake (Fig. 1(c)). This coincidence is consistent with our result that the cause of the observed fast subsidence in the forearc region is the long-term locking of the asperities of the 2011 Tohoku earthquake for several hundreds of years. A similar fast interseismic subsidence on the forearc region has been also observed along the east coast of the Hokkaido in the western Kuril subduction zone^10^, where the gigantic earthquake occurred in the 17th century^13^. We suggest that the fast subsidence at the east coast of Hokkaido is caused by the long-term locking of the asperities of the gigantic earthquake in the 17th century for ~400 years. Therefore, slip deficit of at least 30 m may have accumulated on the asperities of the 17th century event at present.

The interseismic fast subsidence in a forearc region is limited to observations for the NEJP and the western Kuril subduction zones, both of which are subduction zones of an old oceanic lithosphere^7^. Ikeda^7^ proposed that the interseismic fast subsidence in a forearc region and the large slip deficit and afterslip in the deep plate interface are limited to old subduction zones and correlate with the temperature condition of the deep plate interface. We suggest that the temperature conditions of the deep plate interface control the depth location, rheological properties, and development of the shear zone in subduction zones, and therefore, are responsible for the variations in the behavior of the deep plate interface in the subduction zones of various ages.

## Methods

### Model

#### Finite-Element Model

We modeled the crustal deformation by the finite-element method using the GeoFEM code developed at the Research Organization for Information Science and Technology^32^, and Shibazaki *et al*.^33^ modification for viscoelasticity. The model treated the arc-perpendicular two-dimensional deformation assuming a plane strain condition. The model region was 1600 km in arc-perpendicular horizontal length, 800 km in vertical length, and 10 km in arc-parallel length (Fig. 2(a)). The finite-element model comprised 33536 hexahedral elements that were linear Maxwell viscoelastic bodies. The typical size of the elements was 5–10 km for one dimension. The model incorporated the surface topography (land and seafloor topography) and neglected the Earth’s curvature. The finite-element mesh was the same as that in a previous study^34^. Details of the finite-element mesh are given in Supplementary Information. The elastic constants and density for each layer are listed in Supplementary Table S2. The model incorporated the gravity effect caused by the deformation with respect to the initial state^35^. The boundary conditions included free surface on the land surface and seafloor and all sides and the bottom were fixed (Fig. 2(a)). The boundary condition for the plate interface is described in the next subsection.

#### Slip and slip deficit at the plate interface

Our model is based on the backslip model^20^. The backslip model neglects the cumulative long-term crustal deformation owing to steady-state plate subduction; that is, it models only the crustal deformation caused by the perturbation of the slip at the plate interface that is deviated from the long-term plate convergence rate due to interplate coupling, earthquake slip, and afterslip:

1 $${v}_{model}(\xi ,\,t)=v(\xi ,\,t)-{v}_{pl}(\xi )$$

where *v*_*model*_, *v*, and *v*_*pl*_ indicate the slip rate in the backslip model (this study), the actual slip rate, and the long-term steady-state plate convergence rate at the plate interface, respectively. *ξ* indicates the spatial coordinate value along the plate interface and *t* indicates the time. Because the cumulative long-term vertical displacement rate on the NEJP forearc is 0.1–0.2 mm/year and that around the volcanic front is up to 0.6 mm/year^36^, which are one order of magnitude smaller than those in the interseismic period (up to 3–4 mm/year (Fig. 1)), this assumption is reasonable. Kanda & Simons^37^ proposed another kinematic model that considers the effects of long-term plate subduction and bending of the oceanic lithosphere. They concluded that the bending of the oceanic lithosphere causes vertical displacement rates that differ from those predicted by the backslip model by a few tens of percent, and the researchers showed that the two models predict a similar pattern in the displacement rate, except for the area close to the trench. Such differences only require a slight change in the optimum viscosity of the shear zone, which is the fitting parameter of our model, as described later. Therefore, the assumption of the backslip model does not significantly affect the conclusions of this study. We assumed *v*_*pl*_ = 8.35 cm/year from the relative plate motion velocity between the Pacific and North America plates around this region^1^.

During the interseismic period in the model, we applied the backslip on the asperity (Fig. 2(b)) to represent the complete locking, *v*_*model*_ =−*v*_*pl*_, by the dislocation on the plate interface using the split-node method^38^. Locking of the asperity causes the partial slip deficit rate (0 < *v* < *v*_*pl*_) in the surrounding frictionally stable part of the plate interface^11^. The model approximately represents the fault creep on the frictionally stable part (fault creep part) by introducing a thin viscoelastic layer with very low viscosity along the plate interface following Hu *et al*.^21^ (Fig. 2(b,c,f)). We assumed the viscosity and width of the thin viscoelastic layer to be 2.0 × 10^17^ Pa∙s and ~3 km, respectively. We set the viscosity as low as that permitted by the practical calculation time constrained from the required time step. The fault creep part creeps almost freely and its rate varies with time as a mechanical response during the interseismic period.

The division of the asperity and fault creep part on the plate interface was assumed as follows. The time period of the observed vertical displacement that we wanted to explain was from 1882 to 1999. During this period, the asperities of the Miyagi-oki earthquake, which is located at the middle to deeper part of the plate interface, repeatedly ruptured several times^19,29^: 1897 (M7.4^30^), 1936 (M_W_ 7.5^19^), and 1978 (M_W_7.5^19^) (Fig. 1(a)). Therefore, we assumed that the middle to deeper part of the plate interface (14.5–70 km in depth) has been creeping during the interseismic period, as if this is the frictionally stable part (Fig. 2(b)). Therefore, the asperity in the model is the part that is not ruptured in the period and corresponds to the large coseismic slip (>~50 m) area for the 2011 Tohoku earthquake, which is within 75 km of the trench axis^3^ (<14.5 km in depth) (Fig. 2(b)).

For modeling the earthquake cycle, we applied the earthquake slip on the asperity using the split-node method^38^ (Fig. 2(c)) every 600 years. The coseismic slip was 50.1 m, which is equivalent to *v*_*pl*_ multiplied by 600 years. Because our model represents the fault creep part via a thin viscoelastic layer, it takes several years for the earthquake slip to reach the deeper parts of the plate interface. In the earthquake cycle model, we assumed that the locking of asperity begins just after the occurrence of an earthquake and continues to a period just before the occurrence of the next earthquake. We run the earthquake cycles until the cycle reaches a steady state in terms of the surface vertical deformation rate. The spin-up period of our model was the period of the first to fourth earthquake cycles and we show the results of the fifth earthquake cycle.

If we apply the backslip or the earthquake slip only on the asperity, a large stress appears on the tip of the asperity. To avoid this problem, we set the backslip and earthquake slip near the asperity to asymptotically decrease to zero during 50 km from the tip of the asperity. The effect of tapering slip in this way is negligible on the predicted motions at the free surface and within the viscoelastic body.

### Rheological structure

We constructed a two-dimensional heterogeneous rheological structure model for the NEJP island arc-trench system considering the thermal structure, water content, pressure, and lithological units based on the mineral flow laws and typical interseismic strain rates. We briefly describe their essence in this section, and the details are provided in Supplementary Information.

For the thermal structure, water content, and serpentinization in the continental mantle, we used the results of numerical simulations of the mantle corner flow considering dehydration, water transport, and serpentinization by Horiuchi & Iwamori^39^. To constrain the thermal structure in the shallow continental crust (<350 °C), we used D90^40^, which is a deep cutoff depth above which 90% of the crustal earthquakes are located. We used the results of the seismic wave velocity observations and experiments on xenoliths under lower crust conditions in the NEJP^41^ to constrain the temperature in the lower crust under the island arc. We assumed local high temperature column with 10 km width under the volcanic front and considering effect of partial melting on viscosity based on several previous studies^34,41,42^. For the thermal structure of the oceanic lithosphere, we used the thermal structure model by McKenzie *et al*.^43^. The thermal structure below the oceanic lithosphere was assumed to be a mantle adiabatic thermal gradient. We assumed the mantle potential temperature to be 1315 °C^43^ for both the continental and oceanic mantles. We assumed that the continental crust is saturated in water. Water content in the oceanic asthenosphere and background water content in the continental mantle were assumed to be 810 H/Si ppm^44^. Water content in the oceanic lithosphere was assumed to be zero (dry)^44^. If the water content exceeded the saturated solubility of water in olivine^45^, we assumed that the water content in olivine is equivalent to the saturated solubility. The thermal structure, water content, and lithological units to construct the rheological structure of our model are shown in Fig. 2(d–g).

We obtained the viscosity distribution by substituting the thermal structure, water content or water fugacity, pressure, and strain rate to the flow law of minerals. The flow-law parameters of the minerals obtained from rock experiments^46–51^ for each layer in the model are listed in Supplementary Table S3. We calculated the water fugacity using the equation of state in Pitzer & Sterner^52^ following Shibazaki *et al*.^53^. We assumed that the strain rate is 10^−7^/year based on the typical interseismic strain rate in the NEJP region^54^ and constant in time and uniform in space. Assuming the constant strain rate in time means assuming a Newtonian rheology. This assumption overestimates the viscosity early in the postseismic deformation, where the strain rate is much faster, i.e., the deviatoric stress is much larger, than that in the interseismic period. However, we focus on the crustal deformation during the earthquake cycle for long time scales, e.g., a hundred years. Therefore, this assumption does not matter much.

#### Shear zone

Deeper in the plate interface, the behavior shifts from frictional slip on the fault plane to volumetric and ductile deformation in shear zones (e.g., ref.^12^). We assumed that the transition depth from the brittle regime (frictional slip on the fault) to the ductile regime (volumetric ductile deformation in the shear zone) is 70 km (Fig. 2(b,c)) based on the deeper end of the estimated earlier afterslip that occurred ~9 months after the 2011 Tohoku earthquake^34^. However, the viscosity, width, and shape of the shear zone are not well understood. Therefore, we tested different scenarios for the shear zone and selected the optimal one that best explained the observed vertical crustal deformation. The width increased from ~3 km to ~10 km and the viscosity linearly increased from 2 × 10^17^ Pa∙s to 2 × 10^19^ Pa∙s toward the deeper parts (Fig. 2(g)) to represent the transition from localized to distributed deformation^12^.

## Supplementary information

Supplementary Information

## Data Availability

No datasets were generated or analyzed during the current study. Original leveling data can be accessed with permission of the Geospatial Information Authority of Japan. Contact gsi-intex@ml.mlit.go.jp for getting the permission. Original GNSS data of the GEONET F3 solution are available from the Geospatial Information Authority of Japan. Access http://datahouse1.gsi.go.jp/terras/terras_english.html or contact gsi-data@ml.mlit.go.jp.
