# Supplementary material for: Mechanism of subsidence of the Northeast Japan forearc during the late period of a gigantic earthquake cycle
Source: Sci Rep. 2019 Apr 5;9:5726. doi: 10.1038/s41598-019-42169-y (PMC6451026; doi:10.1038/s41598-019-42169-y)
Supplement: Supplementary file 1 — Supplementary Information [file 41598_2019_42169_MOESM1_ESM.docx]

**Supplementary Information of “Mechanism of subsidence of the Northeast Japan forearc during the late period of a gigantic earthquake cycle”**

**Ryohei Sasajima^1,2,*^, Bunichiro Shibazaki^1^, Hikaru Iwamori^3,4,5^, Takuya Nishimura^2^, and Yoshihiko Nakai^1^**

^1^International Institute of Seismology and Earthquake Engineering, Building Research Institute, Tsukuba, 305-0802, Japan.

^2^Disaster Prevention Research Institute, Kyoto University, Uji, 611-0011, Japan.

^3^Earthquake Research Institute, The University of Tokyo, Tokyo, 113-0032, Japan.

^4^Department of Solid Earth Geochemistry, Japan Agency for Marine-Earth Science and Technology, Yokosuka, 237-0061, Japan.

^5^Department of Earth and Planetary Sciences, Tokyo Institute of Technology, Tokyo, 152-8551, Japan.

*Correspondence and requests for materials should be addressed to R. S. (email: r.sasajima@gmail.com).

**Table of contents**

**p. 2 : Modeling co- and post-seismic deformation of major inland earthquakes**

**p. 9 : Time series of the leveling benchmarks**

**p. 11 : Parameter sensitive analysis**

**p. 26 : Velocity field in the model of earthquake cycle**

**p. 28 : Comparison to GNSS data**

**p. 33 : Geometry of the structure**

**p. 34 : Elastic constants and density**

**p. 35 : Details of the methods to construct the rheological structure**

**p. 45 : References for Supplementary Information**

**Modeling co- and post-seismic deformation of major inland earthquakes**

This study focuses on vertical deformation due to interplate coupling and slip on the subduction plate interface. The original leveling data (Fig. 1(a)), however, include vertical deformation caused by large inland earthquakes. To exclude the vertical deformation caused by co- and post-seismic deformation of large inland earthquakes from the leveling data, we calculated the co- and post-seismic deformations of the 1896 Riku-u earthquake (M_W_7.3)^1^, 1964 Niigata earthquake (M_W_7.6)^2^, and 1983 Japan Sea earthquake (M_W_7.85)^3^ as described in the following subsections. The calculated total vertical displacement caused by these three earthquakes in 1900–1993 is shown in Fig. 1(b). We subtracted the calculated vertical displacement from the original vertical displacement data and obtained the corrected vertical displacement rate, which was used in this study, as shown in Fig. 1(c).

*1896 Riku-u earthquake*

The 1896 Riku-u earthquake (M_W_7.3) occurred on August 31, 1896, at 140.5–140.9$^{\circ}$E and 39.3–39.7$^{\circ}$N^1,4^. A survey of the surface ruptures of this earthquake revealed that the 1896 Riku-u earthquake occurred on the Senya Fault, which is an east-dipping reverse fault^4^. The earliest leveling measurement along the leveling route near the epicenter of this earthquake was conducted in 1900^5^. Therefore, the leveling data used in this study herein exclude the coseismic deformation of the 1896 Riku-u earthquake. Thatcher *et al*.^1^ revealed that the postseismic viscoelastic relaxation of the 1896 Riku-u earthquake caused large and long-duration subsidence (up to 35 cm in 1900–1974) near the epicenter obtained from the five leveling measurements in 1900, 1933, 1956, 1966, and 1974^5^. The corresponding local subsidence rate can be observed in the original leveling data of Fig. 1(a). The researchers modeled a postseismic viscoelastic relaxation of the 1896 Riku-u earthquake, assuming a layered elastic and viscoelastic half-space that is an elastic plate (with a thickness of 30 km) overlying a viscoelastic half-space with uniform rigidity of 30 GPa. Results revealed that the spatiotemporal distribution of the vertical displacement observed by the leveling in 1900–1974 can be well explained by the viscoelastic relaxation of the 1896 Riku-u earthquake with a viscosity of 10^19^ Pa·s in the viscoelastic asthenosphere.

On the basis of their study^1^, we calculated the postseismic deformation caused by the viscoelastic relaxation of the 1896 Riku-u earthquake by using the PSGRN/PSCMP code^6^. This code can calculate the elastic and viscoelastic deformation due to seismic sources in a layered elastic and/or viscoelastic half-space considering the effect of gravity, developed by Wang *et al*.^6^ (available at https://github.com/pyrocko/fomosto-psgrn-pscmp (accessed on July 31, 2018)). We utilized the same fault parameters (Supplementary Table S1 and Fig. 1(a)) and model structure (the elastic thickness, rigidity, and viscosity of the asthenosphere were 30 km, 30 GPa, and 10^19^ Pa·s, respectively) used or determined by Thatcher *et al*.^1^ to obtain the same postseismic deformation estimated by their study^1^. We assumed that the density was 3000 Kg/m^3^. We subtracted the calculated postseismic deformation in 1900–1993 from the leveling data shown in Fig. 1(a). After the subtraction, the spatial distribution of the vertical displacement rate around the epicenter becomes simple, that is, subsidence in the forearc and slow uplift in the back arc (Fig. 1(c)).

*1964 Niigata earthquake*

The 1964 Niigata earthquake (M_W_7.6) occurred on June 16, 1964, at 139.0–139.4$^{\circ}$E and 38.0–38.8$^{\circ}$N^2^. The twice leveling and tidal observations before and after the 1964 Niigata earthquake indicated that the coastal area near the source region of the earthquake subsided by up to 20 cm^7^. Two different fault models, namely, west-dipping^7^ and east-dipping^2^ reverse faults, were proposed according to the seismological, leveling, and tidal observations. We assumed the east-dipping reverse fault by Satake & Abe^2^ (Supplementary Table S1 and Fig. 1(a)) based on a recent study on its aftershock relocations^8^. Satake & Abe^2^ also proposed additional small sub-fault to explain the observed seafloor deformation and tilt in Awashima Island on the source region. We neglected the small sub-fault, however, because the researchers concluded that its coseismic deformation hardly affects the leveling data and the existence of this sub-fault has large uncertainty^2^.

We calculated the coseismic elastic and postseismic viscoelastic deformations due to the 1964 Niigata earthquake by using the same method used for the 1896 Riku-u earthquake. In addition, we changed the model structure because the rheological structure of the forearc in the offshore and that under the volcanic front significantly differ from each other (Fig. 2(g)). We assumed that the elastic thickness is 50 km and the viscosity of the asthenosphere is $5\times{10}^{19}$ Pa·s, which is five times as large as that of the 1896 Riku-u earthquake, based on the difference in the rheological structure in the two regions used in this study (Fig. 2(g)). We subtracted the calculated co- and post-seismic deformation on June 16, 1964–January 1, 1993, from the leveling data shown in Fig. 1(a). After the subtraction, the relative subsidence along the coastal line compared to the uplift around the volcanic front nearly disappears (Fig. 1(c)). Note that the large subsidence rate in Niigata City (i.e., near 139.0$^{\circ}$E and 37.9$^{\circ}$N), which is located to the south of the source region of the 1964 Niigata earthquake, was caused by groundwater pumping^9,10^.

*1983 Japan Sea earthquake*

The 1983 Japan Sea earthquake (M_W_7.85) occurred on May 26, 1983, at 138.8–139.5$^{\circ}$E and 40.2–41.3$^{\circ}$N^3^. We used the fault model of Satake^3^, which is composed of two fault planes (Supplementary Table S1 and Fig. 1(a)) estimated from long-period surface waves and tsunamis. Subsequently, we calculated the coseismic elastic and postseismic viscoelastic deformations due to the 1983 Japan Sea earthquake by using the same method and model structure used for the 1964 Niigata earthquake. Finally, we subtracted the calculated co- and post-seismic deformations on May 26, 1983–January 1, 1993, from the leveling data shown in Fig. 1(a). Given that the source region of the 1983 Japan Sea earthquake is far from the region of the leveling benchmarks used in this study (Fig. 1(c)), the effect of co- and post-seismic deformations of this earthquake on the leveling data used in this study is only minimal.

*1894 Shonai and 1914 Senboku earthquakes*

Two other large inland earthquakes that possibly affected the leveling data used in this study are as follows. The first one is the 1894 Shonai earthquake (M7.0) that occurred on October 22, 1894, at 139.9$^{\circ}$E and 38.9$^{\circ}$N^11^ (Fig. 1(a)). Trenching survey and investigation of active faults implied that this earthquake occurred on an east-dipping reverse fault^12^. However, their fault parameters are poorly known. Moreover, almost no surface ruptures are reconized^12^. The earliest leveling measurement along the leveling routes near the epicenter of the 1894 Shonai earthquake was conducted in 1899–1901^5^. Hence, the leveling data exclude the coseismic deformation of the 1894 Shonai earthquake. However, the postseismic deformation 5–7 years after the 1894 Shonai earthquake is probably included in the leveling data near the source region. If this earthquake is on reverse faulting, then subsidence is expected in the postseismic period due to the viscoelastic relaxation similar to the 1896 Riku-u earthquake (Fig. 1(b)). However, the vertical displacement rate of the first period (i.e., 1899–1942) of the leveling data near the source region indicates slightly faster uplift rate in the hanging wall side of the 1894 Shonai earthquake than that of the later periods (i.e., 1942–1956, 1956–1966, and 1966–1973)^5,10^. Thus, the postseismic deformation of the 1894 Shonai earthquake probably did not much affect the leveling data. Therefore, we neglected the postseismic deformation effect of the 1894 Shonai earthquake on the leveling data used in this study. Note that the local large subsidence rate in Sakata City (near 139.8$^{\circ}$E and 38.9$^{\circ}$N; Fig. 1(c)), which is located to the west of the epicenter of the 1894 Shonai earthquake, was caused by groundwater pumping^9^.

The other large inland earthquake is the 1914 Senboku earthquake (M6.5–6.6) that occurred on October 22, 1894, at 140.32$^{\circ}$E and 39.46$^{\circ}$N^13^ (Fig. 1(a)). Recent analysis of the seismic intensity inversion^13^ suggested that the magnitude of the 1914 Senboku earthquake is only 6.5–6.6 and that M7.1 estimated by Utsu^11,14^ is too large. Although the magnitude of this earthquake is only 6.5–6.6, its coseismic deformation might affect the leveling data because the source region is close to the leveling route (Fig. 1(a)). If we assume that the east-dipping pure reverse faulting with the slip of 1.275 m on the 45$^{\circ}$ dipped fault plane is buried at 6­–13 km depth according to Kanda & Takemura^13^, then up to 10 cm of the surface uplift should be observed at several leveling benchmarks. However, the leveling data in 1899–1942 does not indicate significant local uplift near the source region. Hence, this earthquake probably did not cause significant surface vertical displacement at the leveling benchmarks. Thus, we neglected the effect of the 1914 Senboku earthquake on the leveling data used in this study.

|  | M_W_ | Longitude^[1]^ | Latitude^[1]^ | Depth^[1]^ | Length | Width |
| --- | --- | --- | --- | --- | --- | --- |
| [Unit] |  | [$^{\circ}$E] | [$^{\circ}N$] | [km] | [km] | [km] |
| 1896 Riku-u | 7.3 | 140.529 | 39.327 | 0 | 50 | 21.21 |
| 1964 Niigata | 7.6 | 139.011 | 38.061 | 1 | 80 | 30 |
| 1983 Japan Sea^[2]^ | 7.85 | 139.178 | 40.742 | 1 | 60 | 40 |
|  |  | 138.785 | 40.245 | 1 | 60 | 40 |

|  | Strike | Dip | Rake | Slip | References |
| --- | --- | --- | --- | --- | --- |
| [Unit] | [$^{\circ}$] | [$^{\circ}$] | [$^{\circ}$] | [m] |  |
| 1896 Riku-u | 25 | 45 | 90 | 4 | Ref. 1 |
| 1964 Niigata | 9 | 34 | 90 | 3.3 | Ref. 2 |
| 1983 Japan Sea^[2]^ | 340 | 30 | 90 | 4 | Ref. 3 |
|  | 20 | 30 | 90 | 5 |  |

[1] Longitude, latitude, and depth at the upper-left corner of the rectangular fault plane.

[2] Fault model of the 1983 Japan Sea earthquake by Satake^3^ is composed of the two fault planes.

**Supplementary Table S1:** Fault parameters of the three major inland earthquakes for modeling their co- and post-seismic deformations.

**Time series of the leveling benchmarks**

Supplementary Figure S1 shows the time series of vertical displacement at the selected leveling benchmarks relative to the reference point in the backarc (the data is derived from Nishimura^15,16^). Note that these time series were not corrected for the deformation caused by major inland earthquakes, which were corrected for in this study. The vertical velocities remain almost constant over time, except for the effects of the co- and postseismic deformation from the major inland earthquake (1964 Niigata earthquake, M_W_7.6)) and the major interplate earthquakes (e.g., 1978 Miyagi-oki earthquake (M_W_7.5)). The effects of these earthquakes on displacement are relatively small compared to long-term (~100-year) deformation. The effect of the inland earthquake was corrected for in this study. It was not necessary to correct for the effect of the interplate earthquakes because the recurrence interval of the majority of M_W_ ~ 7–8 interplate earthquakes in the study region is shorter than the observation period^17^, so the majority of their co- and postseismic deformations will have been canceled out by interseismic deformation due to locking at their asperities. Therefore, the assumption that the vertical displacement rate during the ~100 years before the 2011 Tohoku earthquake is constant over time is appropriate for this study.

**Supplementary Figure S1:** Time series of the selected leveling benchmarks. (a) Vertical displacement rate in the Northeast Japan (NEJP) island arc obtained through leveling measurements^6^ made during the periods from 1892–1906 to 1986–1999. The map of the vertical displacement rate is adapted from Nishimura^15,16^. Colored squares indicate the selected leveling benchmarks potted in (b). The other details are described in the caption of Fig. 1(a) in the main manuscript. (b) Time series of the selected leveling benchmarks. Vertical displacements are derived from Nishimura^15,16^. Each colored symbol corresponds to data from the leveling benchmarks indicated by the colored square symbols in (a). Vertical displacements are relative to the leveling benchmark indicated by a white square in (a). Gray vertical bars indicate the timing of major earthquakes, whose epicenters or fault planes are shown in (a). Figure was created using the Generic Mapping Tools (GMT, http://gmt.soest.hawaii.edu/)^67^.

**Parameter sensitive analysis**

We tested for the effects of changing the parameters introduced into our model, such as rheological structure.

First, we changed the viscosity of the shear zone. How changing the viscosity of the shear zone changes the result is shown in Supplementary Figs. S2 and S3. The two models shown in Supplementary Fig. S2 are (a) the model in which the viscosity of the shear zone is slightly higher than that of the original model, and (b) the model in which the viscosity of the shear zone is slightly smaller than that in the original model. The viscosity of the shear zone in the original model linearly increased from $\eta_{1}=2\times{10}^{17}$ Pa·s at the shallower edge of the shear zone to $\eta_{2}=2\times{10}^{19}$ Pa·s in the mantle wedge. $\eta_{2}$ for case (a) was $4\times{10}^{19}$ Pa·s and $\eta_{2}$ for case (b) was $1\times{10}^{19}$ Pa·s. $\eta_{1}$ was common for cases (a), (b), and the original model. Therefore, the viscosity of the shear zone in cases (a) and (b) was 1–2 times higher and 1–2 times lower than that in the original model, respectively. The vertical displacement rate on the forearc region is very sensitive to the viscosity of the shear zone. Only twice the difference in viscosity produces substantially different results. The higher viscosity of the shear zone delays the propagation of the slip deficit to the shear zone; consequently, it delays increasing the subsidence rate in the forearc region with the duration of the locking of the asperity. The deformation rate across the shear zone is controlled by the width of the shear zone divided by the viscosity, i.e., there is a trade-off between the width and viscosity of the shear zone. This sensitivity suggests that the vertical displacement rate on the forearc region through an earthquake cycle can constrain the value of the width of the shear zone divided by the viscosity.

Second, we examined the necessity of incorporating the shear zone and the resulting deep slip deficit. Supplementary Figure S3 shows the results with the high-viscosity shear zone ($\eta_{2}=8\times{10}^{19}$ Pa·s) and those with the elastic shear zone (i.e., no shear zone). The model with a higher viscosity shear zone results in a relatively smaller deep slip deficit rate and cannot reproduce the observed forearc subsidence. The ${v_{sd}}/{v_{pl}}$ value, where $v_{sd}$ indicates the slip deficit rate and $v_{pl}$ indicates the long-term plate convergence rate, of this model at 400 years after locking of the asperity begins is 0.3–0.09 at depths of 70–100 km, whereas that of the original model shown in Fig. 3(a) is 0.4–0.2 at depths of 70–100 km. When there is no shear zone in the model, there is no deep slip deficit deeper than 70 km, resulting in rapid forearc uplift. Next, we changed the viscosity of the asthenosphere for both the model with the higher viscosity shear zone and that without the shear zone, in order to examine whether viscoelastic relaxation can reproduce the observed forearc subsidence without deep slip deficit. We changed the viscosity of the asthenosphere by changing the mantle potential temperature, *T_mc_* and *T_mo_*, where *T_mc_* is the potential temperature of the continental mantle and *T_mo_* is that of the oceanic mantle. The original model is *T_mc_* = *T_mo_* = 1315 °C. We tested an additional six viscosity structures; (1) *T_mc_* = *T_mo_* = 1400 °C, (2) *T_mc_* = *T_mo_* = 1250 °C, (3) *T_mc_* = 1400 °C and *T_mo_* = 1315 °C, (4) *T_mc_* = 1315 °C and *T_mo_* = 1400 °C, (5) *T_mc_* = 1250 °C and *T_mo_* = 1315 °C, and (6) *T_mc_* = 1315 °C and *T_mo_* = 1250 °C. These viscosity structures shown in Supplementary Fig. S4. Supplementary Figure S5 shows the results of the models without the shear zone and with different viscosity structures. None of the models without the shear zone (i.e., no deep slip deficit at a >70 km depth) reproduce the observed interseismic forearc subsidence. Supplementary Figure S6 shows the results for the models with the high-viscosity ($\eta_{2}=8\times{10}^{19}$ Pa·s) shear zone and different-viscosity structures. The ${v_{sd}}/{v_{pl}}$ values of these models are 0.3–0.35 at a depth of 70 km and ~0.09 at 100 km. The models in which *T_mc_* > *T_mo_* produce forearc subsidence, however, they cannot explain the observed interseismic forearc subsidence rate. Supplementary Figure S7 shows the results for the models with the slightly high-viscosity ($\eta_{2}=4\times{10}^{19}$ Pa·s) shear zone and different-viscosity structures. The ${v_{sd}}/{v_{pl}}$ values of these models are 0.37–0.4 at a depth of 70 km and 0.13–0.15 at 100 km. The models with *T_mc_* > *T_mo_* reproduce the observed interseismic forearc subsidence rate. Therefore, the deep slip deficits (${v_{sd}}/{v_{pl}}$ of at least 0.35–0.1 for 70–100 km depth) are necessary to reproduce the observed interseismic forearc subsidence rate. The viscoelastic relaxation mechanism alone without a deep slip deficit cannot explain the observations.

Third, we changed the shape of the shear zone, i.e., where the shear zone passes through the viscoelastic mantle wedge. Supplementary Figure S8 shows the vertical displacement rate on the surface in the model of interplate coupling 400 years after the locking of the asperity began. The three models shown in Supplementary Fig. S8 include (a) the model with the original rheological structure, (b) the model in which the shear zone passes through the viscoelastic mantle wedge at shallow depth, and (c) the model in which the shear zone passes through the viscoelastic mantle wedge at greater depth. The viscosity of the shear zone in all three models was the same. Changing the shape of the shear zone does not significantly affect the results of the surface vertical deformation. Such small differences could be minimized by changing the viscosity of the shear zone. Therefore, the observed vertical displacement data could not constrain the shape of the shear zone, and we chose the shape of the shear zone so that the plate interface and the shear zone smoothly (without sharp bend) passes through the viscoelastic mantle wedge.

Fourth, we tested the effect of the high-temperature anomalies and partial melting under the volcanic front. Supplementary Figure S9 compares the results of the vertical displacement rate on the surface in the model of interplate coupling 400 years after the locking of the asperity began. The three models shown in Supplementary Fig. S9 are (a) the model with the original rheological structure, (b) the model excluding the effect of partial melting under the volcanic front, and (c) the model excluding the high-temperature column (low-viscosity column) under the volcanic front. There is almost no effect of partial melting on the results. The high-temperature column under the volcanic front contributes to the uplift around the volcanic front and the result is slightly closer to the observed vertical displacement rate than the results without the high-temperature column. The mechanism of the additional uplift owing to the existence of the low-viscosity column can be explained as follows: the viscoelastic deformation in the low-viscosity column under the volcanic front due to interplate coupling is arc-perpendicular to horizontal shortening and vertical extension. The viscoelastic vertical extension causes the local uplift above the high-viscosity column. This low-viscosity column under the volcanic front is also required to explain the observed postseismic local subsidence around the volcanic front following the 2011 Tohoku earthquake^18,19^.

Fifth, we tested the effect of serpentinization in the mantle wedge on the results. Supplementary Figure S10 shows the vertical displacement rate on the surface 400 years after the locking of the asperity began in the model of interplate coupling with and without serpentinization. The results without serpentinization exhibit larger subsidence rates around the coastal line in the forearc region and the peak of the subsidence shifts to the offshore compared to the model with serpentinization. The results with serpentinization reproduce the peak of the observed subsidence inland of the forearc region.

Sixth, we tested the effect of changing the range of the asperity, for which we only considered the main asperity of the 2011 Tohoku earthquake. The analysis and surveys of the seafloor deformation near the trench before and after the 2011 Tohoku earthquake indicate that a very large amount of slip (>50 m) occurred within ~75 km of the trench^20^. Therefore, we assumed that the range of the asperity in our model is within ~75 km of the trench. In order to examine the effect of the uncertainty of the results obtained by Sun *et al*.^20^ on our results, we tested a model in which the asperity ranges 100 km away from the trench. We also tested a model in which the asperity ranges between 25 and 100 km from the trench. Supplementary Figure S11 shows the results. Changing the range of the asperity does not affect the surface vertical displacement rate of the land area (> 215 km from the trench) substantially; it only affects the vertical displacement rate near the trench.

Seventh, we tested the effect of changing the viscosity of the fault creep part on the results. The viscosity of the fault creep part ($\eta_{fc}$) in the original model is $2\times{10}^{17}$ Pa·s. We tested the models in which $\eta_{fc}=1\times{10}^{17}$ Pa·s and $\eta_{fc}=4\times{10}^{17}$ Pa·s. Supplementary Figure S11 shows the results. Changing the viscosity of the fault creep part does not affect the vertical displacement rate of the surface significantly unless the viscosity is so low that the fault creep part can creep almost freely.

We summarize the results of changing model parameters. The results are very sensitive to the viscosity of the shear zone. None of the models without the shear zone (i.e., without a deep slip deficit) can explain the observed interseismic forearc subsidence rate. The shape (depth position) of the shear zone does not affect the results much. Considering a more realistic structure, such as the high-temperature column under the volcanic front and the serpentinization in the mantle wedge, improves the results, although the effect on the results is small. The increasing subsidence rate in the forearc region with the duration of the locking of the asperity is commonly found regardless of the existence of the low-viscosity column under the volcanic front, partial melting, serpentinization, and the shape of the shear zone. Changing the range or location of the asperity or the viscosity of the fault creep part has little effect on the modeled vertical displacement rate on the surface.

**Supplementary Figure S2:** Vertical displacement rate of the surface in the models of interplate coupling for different viscosities of the shear zone. (a, b) Colored lines in the top panels indicate the surface vertical displacement rate in the model of interplate coupling at each $t$, where $t$ is the time from when the locking of the asperity begins. Black dots indicate the observed vertical displacement rate on the surface in the middle northeast Japan (NEJP) island arc for ~100 years before the 2011 Tohoku earthquake based on leveling data (see Fig. 1 in the main manuscript). Vertical pink dashed line indicates the location of the volcanic front. The bottom panel indicates the viscosity structure of the model, whose results are shown in the upper panel. (a) The model with slightly higher-viscosity shear zone than in the original model. (b) The model with slightly lower-viscosity shear zone than that in the original model. Figure was created using the GMT^67^.

**Supplementary Figure S3:** Vertical displacement rate of the surface in the models of interplate coupling with high viscosity at the shear zone and without the shear zone. (a) The model with a higher viscosity at the shear zone than that of the model shown in Supplementary Fig. S2(a). (b) The model without the shear zone (i.e., the shear zone is elastic). The setup of the plots is as in Supplementary Fig. S2. Figure was created using the GMT^67^.

**Supplementary Figure S4:** Enlarged views of the viscosity structures in different models. (a) The original model, where *T_mc_* = 1315 °C = *T_mo_* = 1315 °C, where *T_mc_* is the mantle potential temperature of the continental mantle and *T_mo_* is that of the oceanic mantle. (b) The model in which *T_mc_* = *T_mo_* = 1400 °C. (c) The model in which *T_mc_* = 1400 °C and *T_mo_* = 1315 °C. (d) The model in which *T_mc_* = 1250 °C and *T_mo_* = 1315 °C. (e) The model in which *T_mc_* = *T_mo_* = 1250 °C. (f) The model in which *T_mc_* = 1315 °C and *T_mo_* = 1400 °C. (g) The model in which *T_mc_* = 1315 °C and *T_mo_* = 1250 °C. Viscosity of the shear zone is for the cases with $\eta_{2}=8\times{10}^{19}$ Pa·s (see text in in this Supplementary Information). Figure was created using the GMT^67^.

**Supplementary Figure S5:** Results of the models lacking the shear zone with different asthenospheric viscosity structures. (a, b) Colored lines in the upper panels show the modeled surface vertical displacement rates at $t=400$ years, where $t$ is the time from the initiation of locking at the asperity begins. The pink line shows the result from the original model. Black dots indicate the observed vertical displacement rate at the surface in the middle of the northeast Japan (NEJP) island arc for ~100 years before the 2011 Tohoku earthquake based on leveling data (see Fig. 1 in the main manuscript). Vertical pink dashed line indicates the location of the volcanic front. The middle panels show the cross section of the model focusing on the plate interface. The bottom panels show the modeled slip deficit rates at $t=400$ years on the plate interface (solid lines) and across the shear zone (dashed lines). The slip deficit rate of 8.35 cm/year corresponds to the full coupling. Figure was created using the GMT^67^.

**Supplementary Figure S6:** Results of the models with the high-viscosity (hard) shear zone for different asthenospheric viscosity structures. The setup of the plots is as in Supplementary Fig. S5. Figure was created using the GMT^67^.

**Supplementary Figure S7:** Results of the models with the slightly high-viscosity (slightly hard) shear zone for different asthenospheric viscosity structures. The setup of the plots is as in Supplementary Fig. S5. Figure was created using the GMT^67^.

**Supplementary Figure S8:** Results for different shapes of the shear zone. (a) The modeled ($t=400$ years) and observed surface vertical displacement rates. The setup of the plots is as in the upper panels of Supplementary Fig. S5. Pink, blue, and green lines correspond to the original model shown in (b), the model in which the shear zone passes through the viscoelastic mantle wedge at shallower parts, as shown in (c), and the model in which the shear zone passes through the viscoelastic mantle wedge at deeper parts, as shown in (d), respectively. (b–d) Viscosity structure of the three models in (a). Figure was created using the GMT^67^.

**Supplementary Figure S9:** Results for different rheological structures under the volcanic front. The setup of the plots is as in Supplementary Fig. S8. (a) Pink, blue, and green lines correspond to the model with the original rheological structure in (b), the model without partial melting in (c), and the model without the high-temperature column under the volcanic front in (d), respectively. Figure was created using the GMT^67^.

**Supplementary Figure S10:** Results for different rheological structures in the serpentinized mantle wedge. The setup of the plots is as in Supplementary Fig. S8. (a) Pink and green lines correspond to the model with serpentinization (original model) shown in (a) and that without serpentinization shown in (b), respectively. Figure was created using the GMT^67^.

**Supplementary Figure S11:** Results of the models for different ranges of the asperity. (a) Modeled ($t=400$ years) and observed surface vertical displacement rates. The setup of the plots is as in the upper panels of Supplementary Fig. S5. Pink, blue, and green lines indicate the results of the original model shown in (c), the model with a larger asperity shown in (d), and the model shown in (e) in which the asperity locates further in the landward direction, respectively. (b) Modeled slip deficit rates at $t=400$ years on the plate interface (solid lines) and across the shear zone (dashed lines). (c–e) Segmentation of the plate interface in the models. Figure was created using the GMT^67^.

**Supplementary Figure S12:** Results of the models with different fault creep part viscosities. (a) Modeled ($t=400$ years) and observed surface vertical displacement rates. The plots are set up as in the upper panels of Supplementary Fig. S5. Pink, blue, and green lines indicate the results of the original model (fault creep part viscosity $\eta_{fc}=2\times{10}^{17}$ Pa·s), the model in which $\eta_{fc}=4\times{10}^{17}$ Pa·s, and the model in which $\eta_{fc}=4\times{10}^{17}$ Pa·s, respectively. (b) Modeled slip deficit rates at $t=400$ years on the plate interface (solid lines) and across the shear zone (dashed lines). Figure was created using the GMT^67^.

**Velocity field in the model of earthquake cycle**

Supplementary Figure S13 shows the cross-section of the modeled velocity vector in the continental crust and mantle in the model of the earthquake cycle. The results at 100, 250, and 600 years after the fifth earthquake are shown.

**Supplementary Figure S13:** Cross-section of the modeled velocity vector in the continental crust and mantle and the modeled vertical surface displacement rate. (a–c) Pink line in the upper part of each image indicates the modeled vertical surface displacement rate ($\dot{U}_{z}$) at (a) *t* = 100 years, (b) *t* = 250 years, and (c) *t* = 600 years, where *t* is the time from the fifth earthquake in the model. Black dots in image (c) indicate the observed $\dot{U}_{z}$ in the middle NEJP island arc for ~100 years before the 2011 Tohoku earthquake based on leveling data (see Fig. 1 in the main manuscript). Colored arrows in the bottom of each image indicate the modeled velocity vectors in the continental crust and mantle at each *t*. The magnitude of the velocity is indicated by the length and color of the arrows. Black lines at the bottom of each image indicate the land surface or seafloor, continental Moho, and upper surface of the subducting oceanic lithosphere. Figure was created using the GMT^67^.

**Comparison to GNSS data**

To confirm the observed interseismic vertical displacement rates from independent observations that have different time scale, we also compared our results with the Global Navigation Satellite System (GNSS) observations before the 2011 Tohoku earthquake. We used data from a dense network of continuous GNSS stations in Japan, GEONET, which are operated by the Geospatial Information Authority of Japan (GSI). We used the daily coordinates of the GEONET F3 solution^21^ provided by the GSI (http://terras.gsi.go.jp, accessed ­­­on February 28th, 2019; see http://datahouse1.gsi.go.jp/terras/terras_english.html for an English-language description of GEONET, accessed on March 7th, 2019). The reference frame of the daily coordinates of the GEONET F3 solution by the GSI is the International Terrestrial Reference Frame (ITRF) 2005^22^. We corrected the artificial offsets of the coordinates caused by such as maintenance of stations using the estimated offset values provided by the GSI (http://terras.gsi.go.jp, accessed on February 28th, 2019).

We used data from May 1997 to May 2005. The 2005 Miyagi-oki earthquake, which was an interplate earthquake (M_W_7.1^23^) (Fig. S14(a)), occurred on August 16th, 2005. We extracted data up to 2005 because significant postseismic deformation was observed at some stations in the region focused on in this study. Although GEONET observations started in 1996, we used post-1997 data because many more stations started operating by that time. There is strong seasonality in the vertical displacement in the Northeast Japan region, particularly in winter, presumably related to snow at some stations. We avoided the seasonal perturbations by obtaining the vertical displacement rates at each station as follows. We averaged the time series of the vertical component during the months of May 1997 and May 2005, respectively. We then calculated the difference in the one-month averaged value for May 1997 and May 2005 and divided it by the time difference between the two periods, i.e., 8 years.

During this period, two earthquakes occurred that significantly affected the vertical displacement of some of the stations in the region focused on in this study. One was an intraslab earthquake in the subducted Pacific slab, near the coastal region of the Miyagi prefecture (M_w_7.0^23^, the centroid depth of 74 km^23^) occurred on May 26th, 2003 (Fig. S14). The other was the 2003 northern Miyagi earthquake, a shallow crustal earthquake (M_w_6.1^23^, the centroid depth of 5 km^23^) occurred on July 26th, 2003 (Fig. S14). GEONET observed no significant post-seismic deformation in the vertical component for these two earthquakes. We used the following procedure to correct for the coseismic vertical displacements caused by these two earthquakes. We took an average of the time series of the vertical component during 30 days before and after each earthquake, respectively. We then calculated the difference between the averaged values. If the calculated difference exceeded the largest standard deviation of the vertical component for 30 days before or after the earthquake, we subtracted the difference from the data to correct for the coseismic vertical displacement. In addition, we subtracted the vertical displacement caused by the postseismic deformation of the three major inland earthquakes from the data, as with the leveling data (see *Modeling co- and post-seismic deformation of major inland earthquakes* section of this Supplementary Information). We calculated the standard deviation of each vertical displacement rate by assuming linear propagation of the standard deviation of the data.

Figure S14(a) shows a map view of the calculated vertical displacement rate based on the corrected GNSS observations. The distribution of the vertical displacement rates is similar to that from leveling for ~93 years (Fig. 1(c) in the main manuscript), except for a few stations that exhibit localized rapid subsidence, presumably due to groundwater pumping or their locations being near the epicenter of the 2004 mid-Niigata earthquake (M_W_6.6^23^, the centroid depth of 74 km^23^). (We did not correct for the vertical displacement caused by the 2004 mid-Niigata earthquake because it did not affect the vertical displacement of stations located in the region focused on in this study). Forearc subsidence at 37.5–39.5$^{\circ}$N and backarc uplift were observed in both the leveling measurements made during 1892–1999 and GNSS observations made during 1997–2005. The uplift along the eastern coastal region at 39.5–40.5$^{\circ}$N is presumably due to postseismic deformation after the 1994 Sanriku-oki earthquake^15^ (M_W_7.7^17^). Figure S14(b) shows a comparison between the observed vertical surface displacement as a function of distance from the trench and the modeled vertical displacement for 600 years after the gigantic earthquake, i.e., just before the gigantic earthquake. The modeled results are roughly consistent with the observations.

Although our results are roughly consistent with GNSS observations, our results cannot strictly be compared to GNSS observations because our model does not include the locking of the moderate-sized asperities (e.g., those involved in the ~M_W_7.5 Miyagi-oki earthquake) in the middle to deeper parts of the plate interface, which did not rupture during 1996–2011 (Fig. 1(a)). The 2005 Miyagi-oki earthquake (M_W_7.1^23^) only ruptured a small proportion of the ~M_W_7.5 Miyagi-oki earthquake asperities^24^. However, tidal observations near the epicenter of the 1978 Miyagi-oki earthquake (M_W_7.5) indicate that the coastal region subsided by 4–8 cm at the 1978 Miyagi-oki earthquake and almost no vertical deformation occurred in the following ~10-year postseismic period, probably due to postseismic deformation. This differed from other periods, when the coastal region subsided at a rate of ~4 mm/year^16^. Therefore, the coseismic subsidence and the postseismic absence of subsidence almost canceled each other out near the coastal area. The sum of the coseismic and postseismic deformations due to the ~M_W_7.5 Miyagi-oki earthquake should be equivalent to the interseismic deformation rate due to the locking of the asperities involved multiplied by the recurrence interval (~40 years), and this means that the locking of these asperities did not significantly affect vertical displacement near the coastal region. This can also be confirmed by the leveling data time series, which indicates that the 1978 Miyagi-oki earthquake did not significantly affect the overall tendency in subsidence over ~93 years (Fig. S1(b)). Thus, we can roughly compare our results to the vertical displacement rate observed by the GNSS before the 2011 Tohoku earthquake.

Unlike with the vertical displacement, we cannot strictly compare our results to the horizontal displacement observed by the GNSS observations because the locking of the asperities involved in the ~M_W_7.5 Miyagi-oki earthquake systematically enhances the landward horizontal motion in the land area, although its effect may be relatively smaller than the locking of the main asperities of the 2011 Tohoku earthquake. Therefore, our model, which neglects the ~M_W_7.5 Miyagi-oki earthquake asperities, may systematically slightly underestimate the landward horizontal displacement rates compared to those observed. Therefore, we can make only a very rough comparison. However, our results, which show landward horizontal displacement rates of 3.0–3.7 cm/year before the gigantic earthquake, are roughly consistent with those observed by GNSS for 1997–2000 (3–4 cm/year)^16^, as mentioned in the main manuscript.

**Supplementary Figure S14:** Vertical displacement rates in Northeast Japan observed by GNSS and comparison to the modeled results. (a) Vertical displacement rates in Northeast Japan from GNSS observations from May 1997 to May 2005, corrected for the effect of some intraplate earthquakes (see text). The contour interval is 0.2 cm/year. Gray and black dots indicate GNSS stations. Stations indicated by red arrows have anomalous local subsidence, probably due to groundwater pumping. Black circles, red squares, and the red star indicate the interplate earthquakes^23^, inland crustal earthquakes^23^, and intraslab earthquake^23^ mentioned in the *Comparison to GNSS data* section of Supplementary Information, respectively. The meaning of the other symbols is the same as in Fig. 1(a) in the main manuscript. (b) Comparison between the observed and modeled vertical displacement rate. Black squares indicate vertical displacement rates based on the GNSS data indicated by black dots bounded by two arc-perpendicular lines in Fig. 1(a). The pink line indicates the modeled vertical displacement rate 600 years from the gigantic earthquake (i.e., just before the gigantic earthquake). Error bars indicate 1$\sigma$ uncertainties. The vertical pink dashed line indicates the location of the volcanic front. Data indicated by the red arrow is from the stations with anomalous local subsidence, probably due to groundwater pumping, shown in Fig. S14(a). Figure was created using the GMT^67^.

**Geometry of the structure**

The geometry of the structure in our finite-element model is as follows. The oceanic slab geometry was based on seismic reflection surveys^25,26^ and seismic tomography^27^. The geometry of the Conrad discontinuity, which is the boundary between the continental upper crust and lower crust, and Moho discontinuity, which is the boundary between the continental lower crust and mantle, was based on the seismic reflection surveys^28,29^. Supplementary Figure S15 shows the geometry of the Conrad, Moho, and oceanic slab and the layer division for the elastic constants, density, and the minerals used to construct the viscosity structure in our finite-element model. The finite-element mesh was the same as that in a previous study^19^.

**Supplementary Figure S15:** Geometry of the surface, Conrad, Moho, and slab and layer division in the finite-element model. (a) Enlarged view of the finite-element model focused on the island arc region. The brown and blue lines indicate the upper surface of the continental lithosphere and the upper surface of the oceanic lithosphere/slab, respectively. Green, red, and pink lines indicate the Conrad, Moho, and oceanic Moho, respectively. CUC: Continental upper crust. CLC: Continental lower crust. (b) Whole map view of the finite-element model. Figure was created using the GMT^67^.

**Elastic constants and density**

The Young’s modulus, rigidity, and density for each layer in our finite element model are listed in Supplementary Table S2. For the elastic constants and density in the continental upper and lower crusts, we used seismic wave velocities of the northeast Japan (NEJP) island arc estimated by a study on seismic tomography^30^ ($V_{P}$ = 6.04 km/s and $V_{S}$ = 3.56 km/s at 10 km depth for the upper crust; $V_{P}$ = 6.61 km/s and $V_{S}$ = 3.76 km/s at 25 km depth for the lower crust; $V_{P}$ and $V_{S}$ are the P- and S-wave velocities) and the typical density values for the forearc upper crust (2700 kg/m^3^) and the lower crust (2850 kg/m^3^) of the NEJP island estimated by seismic reflection/refraction and gravity anomalies^31^. For the elastic constants and density in the continental mantle, we used seismic wave velocities for the NEJP island arc estimated by seismic tomography^30^ ($V_{P}$ = 8.27 km/s and $V_{S}$ = 4.56 km/s at 120 km depth) and a density value (3354 kg/m^3^ at 122.6 km depth) from the 1D Reference Earth Model “STW105”^32^.

For the elastic constants and density in the oceanic crust, we used the P-wave velocity for global oceanic crust ($V_{P}$ = 6.9 km/s at 3 km depth below the plate upper surface) by the seismic wave velocity study^33^, the mean Poisson’s ratio for oceanic crust (0.28)^34^, and a conversion equation from $V_{P}$ to density for oceanic crust by Carlson & Raskin^35^. For the elastic constants and density in the oceanic lithosphere and oceanic mantle, we used $V_{P}$ = 8.008 km/s, $V_{S}$ = 4.494 km/s, and a density of 3354 Kg/m^3^ at 122.6 km depth from the 1D Reference Earth Model “STW105”^32^.

| Layer^[1]^ | Young’s modulus^[2]^ | Rigidity^[2]^ | Density^[3]^ | References |
| --- | --- | --- | --- | --- |
| CUC | 84.3 | 34.1 | 2700 | Refs. 30 and 31 |
| CLC | 101.6 | 40.3 | 2850 | Refs. 30 and 31 |
| Continental mantle | 178.8 | 69.7 | 3354 | Refs. 30 and 32 |
| Oceanic crust | 109.6 | 42.8 | 2942 | Refs. 33–35 |
| Oceanic mantle | 172.1 | 67.7 | 3354 | Ref. 32 |

**Supplementary Table S2:** List of elastic constants and density for each layer in the finite-element model.

[1] CUC: Continental upper crust, CLC: Continental lower crust.

[2] Unit is GPa.

[3] Unit is kg/m^3^.

**Details of the methods to construct the rheological structure**

We constructed a two-dimensional heterogeneous rheological structure model for the NEJP island arc-trench system by substituting the thermal structure, water content or water fugacity, pressure, and strain rate to the flow law of minerals obtained from rock experiments.

*Flow law*

For the ductile deformation, we only considered dislocation creep, which is the dominant mechanism for high stress or strain rate^36^, and neglected diffusion creep. The flow law of the dislocation creep can be expressed as (e.g., ref. ^36^)

$${\dot{\varepsilon}'}_{E}^{(v)}=D{{\tau'}_{E}}^{n} (1)$$

$$D=\frac{3^{\frac{n+1}{2}}}{2}AW^{r}\exp\left( -\frac{E^{*}+PV^{*}}{R_{g}T} \right) (2)$$

where $A$*, n, r,* $E^{*}$*,* $V^{*}$*, P,* $R_{g}$, and *T* are the pre-exponential factor, exponent for differential stress, exponent of water content or water fugacity, activation energy, activation volume, pressure, gas constant, and temperature, respectively. ${\dot{\varepsilon}'}_{E}^{(v)}$ and ${\tau'}_{E}$ indicate the second invariant of the viscous deviatoric strain rate tensor and the second invariant of the deviatoric stress tensor, respectively. Factor $\frac{3^{\frac{n+1}{2}}}{2}$ converts the axial rock experimental conditions to the invariant tensor form (arbitral deformation condition)^36^. $W$ is the water content for olivine and the water fugacity for quartz and anorthite, in which we assume minerals saturated in water. We calculated *P* by the total mass above each point using a gravitational acceleration of 9.85 m/s^2^. When we calculated *P*, we included a seawater layer, whose density was assumed to be 1050 kg/m^3^, although our finite element model did not include a seawater layer. We calculated the water fugacity from *T* and *P* by using the equation of state in Pitzer & Sterner^37^ following Shibazaki *et al*.^38^. The flow parameters of minerals based on experiments^39–44^ for each layer of our model are listed in Supplementary Table S3.

From the definition of viscosity—$\eta=\frac{{\tau'}_{E}}{2\dot{\varepsilon'}_{E}^{\left( v \right)}}$—and equations (1) and (2), the effective viscosity owing to dislocation creep is as follows:

$\eta=\frac{1}{2}D^{-\frac{\left. 1 \right.}{n}}{\dot{\varepsilon'}_{E}^{\left( v \right)}}^{\frac{1-n}{n}}$. (3)

We converted the nonlinear rheology to linear viscosity (a Newtonian viscoelasticity) by assuming $\dot{\varepsilon'}_{E}^{\left( v \right)}$ constant in time. We also assumed uniform $\dot{\varepsilon'}_{E}^{\left( v \right)}$ and equivalent to the typical strain rate of the interseismic crustal deformation (${10}^{-7}$[/year] of the axial strain rate^45^, i.e., $\dot{\varepsilon'}_{E}^{\left( v \right)}=0.5\times{10}^{-7}$ [/year]).

*Thermal Structure*

For the thermal structure in the continental mantle, we used the results of numerical simulations of the mantle corner flow considering dehydration, water transport, and serpentinization simulated by Horiuchi & Iwamori^46^ (hereinafter HI16). We adjusted the mantle potential temperature to 1315 °C, which is the same as that of the oceanic mantle in this model according to McKenzie *et al*.^47^. For the deep parts (>300 km) of the continental mantle, we extrapolated the thermal structure assuming an adiabatic thermal gradient. The mantle adiabatic thermal gradient was calculated as follows:

$$\frac{dT}{dz}\left( z \right)=\frac{\alpha\left( T,P \right)g\left( z \right)T\left( z \right)}{C_{p}\left( T \right)}, (4)$$

where *z*, *T*, *P*, $\alpha$, *g*, and $C_{p}$ indicate depth, temperature, pressure, volumetric thermal expansion coefficient, gravitational acceleration, and isobaric heat capacity, respectively. We calculated the temperature and pressure dependent $\alpha$ of olivine (Fo_90_Fa_10_) following Bouhifd *et al*.^48^ and Hacker & Abers^49^. We used the temperature dependent $C_{p}$ of olivine (Fo_100_) by Berman & Aranovich^50^. We used the depth-dependent gravitational acceleration based on the Preliminary Reference Earth Model^51^.

To constrain the thermal structure in the shallow continental crust, we used D90, which is a deep cutoff depth above which 90% of the crustal earthquakes are located. D90 in the crust is known to correspond to 300–400 °C based on the geothermal gradient data (e.g., ref. ^52^). Therefore, we assumed that the thermal structure of the continental crust above D90 is linear from 0 °C at the surface to 350 °C at D90. We calculated D90 using the relocated continental intraplate earthquakes by Omuralieva *et al*.^52^. The grid size required for calculating D90 was 0.3*°* by 0.3*°*, and we calculated D90 every 0.05*°* of the center of the grid if the number of earthquakes in the grid was equal or greater than 30; this is the same as in Omuralieva *et al*.^52^. We projected the calculated D90 within the study region (Supplementary Fig. S16(a)) that is the same as the defined range of the leveling data used in this study to the distance from the trench and obtained D90 as a function of the distance from the trench by taking 10 km as the moving average (Supplementary Fig. S16(b)). We used this D90 as a function of the distance from the trench to construct the thermal structure of the shallow part. Because the depth precision of offshore events is not good, D90 around the Japan Sea coastal region is overestimated. Therefore, we assumed the D90 of the Japan Sea coastal region and under the Japan Sea (>352 km from the trench) as 16 km, which is the D90 under Sado Island (Supplementary Fig. S16(a)). This assumption is reasonable because the heat flow on Sado Island represents the typical heat flow under the Japan Sea^53^. For the Pacific Ocean side (<235 km from the trench), we used the thermal structure simulated by HI16 at all depths of the hanging wall. We chose the shifting point (235 km from the trench) such that the thermal structure constructed using D90 and that simulated by HI16 were naturally tied to each other.

Under the inland region of the island arc, we linearly interpolated the thermal structure in the vertical direction between 350 °C constrained by D90 and 1100 °C simulated by HI16. The 1100 °C temperature was chosen so that the temperature around the Moho depth becomes ~800 °C, which was constrained by the seismic wave velocity observations and experiments on xenoliths under lower crust conditions in this region^54^. Under the Japan Sea, there is no information about the thermal structure of the lower crust or Moho depth. Therefore, we linearly extrapolated the thermal gradient of the shallow part by D90 up to 1100 °C, and linearly interpolated between 1100 °C and 1200 °C simulated by HI16. We interpolated these two methods at 335–375 km from the trench so that there was no discontinuity in the constructed thermal structure.

The exception for applying the thermal structure described above is under the volcanic front, where locally an extremely high geothermal gradient (>100 °C/km) was observed^53^. Such local extremely high-temperature regions cannot be identified using D90, which has a resolution greater than 30 km. Therefore, under the volcanic front (10 km width), we assumed that the temperature linearly increases from 0 °C at the surface to 650 °C, which is close to the wet solidus of granite^55^, at 10 km depth. For the column deeper than 10 km, we linearly interpolated the temperature in the vertical direction between 650 °C and 1000 °C at the Moho based on the temperature estimates for the lower crust under the volcanic front in this region^54^. We linearly interpolated the temperature between 1000 °C at the Moho and 1100 °C in the mantle wedge simulated by HI16. The local high-temperature (low-viscosity) column under the volcanic front is necessary to explain the observed postseismic deformation of the 2011 Tohoku earthquake around the volcanic front^18,19^.

For the thermal structure of the oceanic lithosphere before it subducts, we used thermal structure model by McKenzie *et al*.^47^—we modeled the thermal structure using the same method and parameters as those of their study. For the thermal structure of the subducted part of the oceanic slab, we calculated the temperature evolution by using the same method under the boundary conditions that the temperature on the slab upper surface was obtained from HI16 and that on the slab lower surface was equivalent to the mantle adiabatic temperature. We referred to another study of a numerical simulation^56^ for the slab upper surface temperature deeper than 300 km. The thermal structure in the oceanic mantle below the oceanic lithosphere and slab was assumed to be the mantle adiabatic thermal gradient.

*Water content*

For the water content in the mantle wedge, we used the results of the numerical simulation in HI16. The background water content in the continental mantle was fixed at 810 H/Si ppm^57^. We assumed that the minerals in the continental crust are saturated in water and the minerals in the oceanic crust and serpentinized mantle wedge are under the constant wet condition in their lock experiment of the ductile flow law. For the water content in the oceanic mantle, we assumed that it is 0 H/Si ppm within 70 km from the oceanic plate’s upper surface for water-depleted oceanic lithosphere^57^ and linearly increases to 810 H/Si ppm in the 70–80 km distance from the oceanic plate’s upper surface^57,58^. If the water content exceeded the saturated solubility of water in olivine^59^, we assumed that the water content in olivine is equivalent to the saturated solubility.

*Lithological units*

We assumed that the minerals in the continental upper crust, continental lower crust, and continental upper mantle are quartz, anorthite, and olivine, respectively. Based on HI16, we assumed that the mineral is antigorite for the serpentinized mantle wedge. The range of the serpentinized mantle wedge was basically based on HI16 as follows. The depth is less than 150 km and the temperature is lower than the upper limit of the temperature at which serpentine can exist stably based on the phase diagram^60^ following the simulated results of HI16 and is higher than 200 °C, which roughly corresponds to the temperature of the trench-side end of the serpentinized part in HI16. Abers *et al*.^61^ suggested that the percentage of the serpentinization in the mantle wedge in the NEJP region is a maximum of 20 %. However, HI16 suggested that a significant viscosity reduction may occur even in case of partially serpentinized parts if the serpentinized weak material forms a network connected along the fluid paths^46^. Therefore, we assumed that the whole of the serpentinized mantle wedge exhibited the viscosity of antigorite. We additionally verified a case in which there was no serpentinization (Supplementary Fig. S10).

We assumed that the minerals in the oceanic crust above 100 km depth and below 100 km depth are gabbro and eclogite, respectively. The 100 km depth approximately corresponds to the theoretical depth of the dehydration reactions in the subducting slab in this region^62^. We assumed that the mineral in the oceanic upper mantle (both oceanic lithosphere, slab and oceanic upper mantle shallower than 410 km) is olivine. The deep upper mantle (>410 km and $\leq$660 km) and lower mantle (>660 km) mainly comprise spinel and perovskite^63^, respectively, which do not well-constrain the flow laws. Therefore, we directly assumed the viscosity for the >410 km depth as the depth profile of the mantle viscosity estimated from the sinking rate of the subducting oceanic slabs^64^.

We considered partial melting of the continental crust under the volcanic front as follows. The viscosity of the continental upper crust deeper than 10 km (>650 °C) was assumed to be ${10}^{18}$ Pa·s based on the previous study of the postseismic deformation of the 2011 Tohoku earthquake around the volcanic front^19^. Nishimoto *et al*.^54^ estimated the melt fraction of the hot continental lower crust (>900 °C) under the volcanic front in this region at 0.4–8.8% v/v. Several percentages of the melt in crustal rocks decreased the viscosity to several tens percent (e.g., ref. ^65^). Therefore, we multiplied the viscosity of the lower crust hotter than 900 °C by 0.25. We neglected the partial melting of peridotite in the mantle wedge (0–6% v/v of melting degree was simulated by HI16) because a few percentages of the melt in peridotite do not decrease the viscosity (for dislocation creep of olivine) much^66^.

| Layer^[1]^ | Mineral | $A$^[2]^ | $n$^[3]^ | $r$^[4]^ | $E^{*}$^[5]^ | $V^{*}$^[6]^ | Reference |
| --- | --- | --- | --- | --- | --- | --- | --- |
| CUC | Quartz | 1.17 × 10^−5^ | 2.97 | 1 | 242 | 0 | Ref. 41 |
| CLC | Anorthite | 1.58 | 3 | 1 | 345 | 38 | Ref. 43 |
| CM, OM | Olivine (wet)^[7]^ | 3.63 | 3 | 1.2 | 410 | 11 | Ref. 40 |
|  | Olivine (dry)^[7]^ | 1.26×10^6^ | 3 | 0 | 510 | 14 | Ref. 40 |
| SPT | Antigorite | 2.82 × 10^−15^ | 3.8 | 0 | 890 | 3.2 | Ref. 44 |
| OC1 | Gabbro^[8]^ | 1.02 × 10^7^ | 3.97 | 0 | 556 | 0 | Ref. 42 |
| OC2 | Eclogite | 2.00 × 10^3^ | 3.4 | 0 | 480 | 0 | Ref. 39 |

[1] CUC: Continental upper crust, CLC: Continental lower crust, CM: Continental mantle, OM: Oceanic mantle, SPT: Serpentinized mantle wedge, OC1: Oceanic crust (<100 km depth), OC2: Oceanic crust ($\geq$100 km depth).

[2] $A$: pre-exponential factor. Unit for stress and water fugacity is MPa, and that for water content is ppm H/Si.

[3] $n$: Exponent for differential stress.

[4] $r$: Exponent for water fugacity for quartz and anorthite or exponent for water content in wet olivine. No water fugacity or water content dependency for antigorite, gabbro, and eclogite was investigated in the experiments and the flow law was obtained under wet conditions.

[5] $E^{*}$: Activation energy. Unit is kJ/mol.

[6] $V^{*}$: Activation volume. Unit is 10^−6^ m^3^/mol. No pressure dependency for quartz, gabbro, and eclogite was investigated in the experiments.

[7] The flow law is described as $\dot{\varepsilon}^{(v)}=\dot{\varepsilon}_{dry}^{(v)}+\dot{\varepsilon}_{wet}^{(v)}$, where $\dot{\varepsilon}^{(v)}$, $\dot{\varepsilon}_{dry}^{(v)}$, and $\dot{\varepsilon}_{wet}^{(v)}$ indicate the total viscous strain rate, viscous strain rate for dry minerals, and viscous strain rate for wet minerals, respectively^40^.

[8] Anorthite 50% diopside 50%^42^.

**Supplementary Table S3:** Flow-law parameters of the dislocation creep in minerals based on experiments.

**Supplementary Figure S16:** D90 in NEJP island arc. (a) The estimated D90 in the NEJP island arc using the relocated foci of crustal earthquakes by Omuralieva *et al*.^52^. Thick and thin contour intervals are 10 and 2 km, respectively. Gray area is the area where D90 is unreliably estimated owing to the small number of earthquakes. Blue line indicates the Japan Trench. (b) D90 projected to the distance from the trench. Black dots indicate the traced D90 in each grid in the area segmented by the two black lines in (a) that are orthogonal to the trench. Pink line indicates the averaged D90, which was obtained by taking 10 km as the moving average, used to construct the thermal structure in the model. Green line indicates the assumed D90 under the Japan Sea region from the typical value of D90 under Sado Island in (a). Figure was created using the GMT^67^.

**References for Supplementary Information**

1. Thatcher, W., Matsuda, T., Kato, T. & Rundle, J. B. Lithospheric loading by the 1896 Riku-u Earthquake, northern Japan: Implications for plate flexure and asthenospheric rheology. *J. Geophys. Res.* **85**(B11), 6429–6435 (1980).

2. Satake, K. & Abe, K. A fault model for the Niigata, Japan, earthquake of June 16, 1964. *J. Phys. Earth* **31**(3), 217–223 (1983).

3. Satake, K. The mechanism of the 1983 Japan Sea earthquake as inferred from long-period surface waves and tsunamis. *Phys. Earth Planet. Int.* **37**(4), 249–260 (1985).

4. Matsuda, T., Yamazaki, H., Nakata, T., & Imaizumi, T. The surface faulting associated with the Riku-u earthquake of 1896. (in Japanese) *Bull. Earthquake Res. Inst. Univ. Tokyo* **55**(3), 795–855 (1980).

5. Kato, T. Crustal movements in the Tohoku district, Japan, during the period 1900–1975, and their tectonic implications. *Tectonophysics* **60**(3), 141–167 (1979).

6. Wang, R., Lorenzo-Martín, F., & Roth, F. PSGRN/PSCMP—a new code for calculating co- and post-seismic deformation, geoid and gravity changes based on the viscoelastic-gravitational dislocation theory. *Comput. Geosci.* **32**(4), 527–541 (2006).

7. Abe, K. Re-examination of the fault model for the Niigata earthquake of 1964. *J. Phys. Earth* **23**(4), 349–366 (1975).

8. Hurukawa, N. & Harada, T. Fault plane of the 1964 Niigata earthquake, Japan, derived from relocation of the mainshock and aftershocks by using the modified joint hypocenter determination and grid search methods. *Earth Planets Space* **65**(12), 1441–1447 (2013).

9. Danbara, T. Synthetic vertical movements in Japan during the recent 70 years. (in Japanese with English abstract) *J. Geod. Soc. Jpn.* **17**(3), 100–108 (1971).

10. Kunimi, T. *et al.* Vertical crustal movements in Japan estimated from the leveling observations data for the past 100 years. (in Japanese) *J. Geograph. Surv. Inst.* **96**, 23–37 (2001).

11. Utsu, T. A list of deadly earthquakes in the World: 1500–2000. In: Lee, W. K., Kanamori, H., Jennings, P. C. & Kisslinger, C. (Eds.), *International Handbook of Earthquake and Engineering Seismology Part A*, 691–717 (Academic Press, San Diego, California, 2002).

12. Suzuki, Y., Ikeda, Y., Watanabe, M., Sugai, T. & Yonekura, N. Holocene activity of the eastern boundary fault of the Shonai plain, Northeast Japan, and its implications for the Shonai earthquake of 1894 —Excavation of the Kannonji Fault—. (in Japanese with English abstract) *J. Seismol. Soc. Jpn.* *2*, **42**, 151–159 (1989).

13. Kanda, K. & Takemura, M. Short-period seismic wave radiation area and magnitude of the 1914 Akita-Senboku earthquake inferred from seismic intensity data by comparison with the 1896 Rikuu earthquake. (in Japanese with English abstract) *J. Seismol. Soc. Jpn.* *2*, **63**, 207–221 (2011).

14. Utsu, T. Seismicity of Japan from 1885 through 1925 — A new catalogue of earthquakes of M>6 felt in Japan and smaller earthquakes which caused damage in Japan —. (in Japanese) *Bull. Earthquake Res. Inst., Univ. Tokyo* **54**(2), 253–308 (1979).

15. Nishimura, T. Crustal deformation of northeastern Japan based on geodetic data for recent 120 years. (in Japanese with English abstract) *J. Geol. Soc. Jpn.* **118**(5), 278–293 (2012).

16. Nishimura, T. Pre-, co-, and post-seismic deformation of the 2011 Tohoku-oki earthquake and its implication to a paradox in short-term and long-term deformation. *J. Disaster Res.* **9**(3), 294–302 (2014).

17. Yamanaka, Y. & Kikuchi, M. Asperity map along the subduction zone in northeastern Japan inferred from regional seismic data. *J. Geophys. Res.* **109**(B7), B07307 (2004).

18. Hu, Y., Bürgmann, R., Freymueller, J. T., Banerjee, P. & Wang, K. Contributions of poroelastic rebound and a weak volcanic arc to the postseismic deformation of the 2011 Tohoku earthquake. *Earth Planets Space* **66**, 106 (2014).

19. Muto, J. *et al*. Heterogeneous rheology controlled postseismic deformation of the 2011 Tohoku-Oki earthquake. *Geophys. Res. Lett.* **43**(10), 4971–4978 (2016).

20. Sun, T., Wang, K., Fujiwara, T., Kodaira, S. & He, J. Large fault slip peaking at trench in the 2011 Tohoku-oki earthquake. *Nat. Commun.* **8**, 14044, 10.1038/ncomms14044 (2017).

21. Nakagawa, H. *et al*. Development and validation of GEONET new analysis strategy (Version 4). *J. Geogr. Surv. Inst.* (in Japanese) **118**, 1–8 (2009).

22. Altamimi, Z., Collilieux, X., Legrand, J., Garayt, B. & Boucher, C. ITRF2005: A new release of the International Terrestrial Reference Frame based on time series of station positions and Earth Orientation Parameters. *J. Geophys. Res.* **112**, B09401 (2007).

23. F-net centroid-moment tensor (CMT) catalog in Japan. National Research Institute for Earth Science and Disaster Resilience (NEID). Available at http://www.fnet.bosai.go.jp/event/search.php?Lang=en (accessed on March 11th, 2019).

24. Okada, T. *et al*. The 2005 M7.2 MIYAGI‐OKI earthquake, NE Japan: Possible rerupturing of one of asperities that caused the previous M7.4 earthquake. *Geophys. Res. Lett.* **32**, L24302 (2005).

25. Ito, A. *et al.* Bending of the subducting oceanic plate and its implication for rupture propagation of large interplate earthquakes off Miyagi, Japan, in the Japan Trench subduction zone. *Geophys. Res. Lett.* **32**(5), L05310 (2005).

26. Takahashi, N. *et al*. Seismic structure and seismogenesis off Sanriku region, northeastern Japan. *Geophys. J. Int.* **159**(1), 129–145 (2004).

27. Nakajima, J. & Hasegawa, A. Anomalous low-velocity zone and linear alignment of seismicity along it in the subducted Pacific slab beneath Kanto, Japan: Reactivation of subducted fracture zone? *Geophys. Res. Lett.* **33**(16), L16309 (2006).

28. Iwasaki, T. *et al*. Extensional structure in Northern Honshu Arc as inferred from seismic refraction/wide-angle reflection profiling. *Geophys. Res. Lett.* **28**(12), 2329–2332 (2001).

29. Nishisaka, H. *et al*. Crustal structure of the Yamato Basin and the margin of the Northeastern Japan Sea using ocean bottom seismographs and controlled sources. (in Japanese with English abstract) *J. Seismol. Soc. Jpn.* *2*, **54**, 365–379 (2001).

30. Nakajima, J., Matsuzawa, T., Hasegawa, A. & Zhao, D. Three-dimensional structure of V_p_, V_s_, and V_p_/V_s_ beneath northeastern Japan: Implications for arc magmatism and fluids. *J. Geophys. Res.* **106**(B10)*,* 21843–21857 (2001).

31. Miura, S. *et al*. Structural characteristics off Miyagi forearc region, the Japan Trench seismogenic zone, deduced from a wide-angle reflection and refraction study. *Tectonophysics* **407**(3–4), 165–188 (2005).

32. Kustowski, B., Ekström, G., & Dziewoński, A. M. Anisotropic shear-wave velocity structure of the Earth's mantle: A global model. *J. Geophys. Res.* **113**(B6), B06306 (2008).

33. White, R. S., McKenzie, D. & O'Nions, R. K. Oceanic crustal thickness from seismic measurements and rare earth element inversions. *J. Geophys. Res.* **97**(B13), 19683–19715 (1992).

34. Shaw, P. R. Age variations of oceanic crust Poisson's ratio: Inversion and a porosity evolution model. *J. Geophys. Res.* **99**(B2), 3057–3066 (1994).

35. Carlson, R. L. & Raskin, G. S. Density of the ocean crust. *Nature* **311**, 555–558 (1984).

36. Karato, S. *Deformation of Earth Materials: An Introduction to the Rheology of the Solid Earth*. (Cambridge Univ. Press, Cambridge, 2008).

37. Pitzer, K. S. & Sterner, S. M. Equations of state valid continuously from zero to extreme pressures for H_2_O and CO_2_. *J. Chem. Phys.* **101**(4), 3111–3116 (1994).

38. Shibazaki, B. *et al.* Heterogeneous stress state of island arc crust in northeastern Japan affected by hot mantle fingers. *J. Geophys. Res. Solid Earth* **121**(4), 3099–3117 (2016).

39. Jin, Z.-M., Zhang, J., Green, II. H. W., & Jin, S. Eclogite rheology: Implications for subducted lithosphere. *Geology* **29**(8), 667–670 (2001).

40. Karato, S. & Jung, H. Effects of pressure on high-temperature dislocation creep in olivine. *Philos. Mag.* **83**(3), 401–414 (2003).

41. Rutter, E. H. & Brodie, K. H. Experimental intracrystalline plastic flow in hot-pressed synthetic quartzite prepared from Brazilian quartz crystals. *J. Struct. Geol.* **26**(2), 259–270 (2004).

42. Dimanov, A. & Dresen, G. Rheology of synthetic anorthite-diopside aggregates: Implications for ductile shear zones. *J. Geophys. Res.* **110**(B7), B07203 (2005).

43. Rybacki, E., Gottschalk, M., Wirth, R. & Dresen, G. Influence of water fugacity and activation volume on the flow properties of fine-grained anorthite aggregates. *J. Geophys. Res.* **111**(B3), B03203 (2006).

44. Hilairet, N. *et al.* High-pressure creep of serpentine, interseismic deformation, and initiation of subduction. *Science* **318**(5858), 1910–1913 (2007).

45. Miura, S. *et al.* Strain concentration zone along the volcanic front derived by GPS observations in NE Japan arc. *Earth Planets Space* **56**(12), 1347–1355 (2004).

46. Horiuchi, S. & Iwamori, H. A consistent model for fluid distribution, viscosity distribution, and flow-thermal structure in subduction zone. *J. Geophys. Res. Solid Earth* **121**(5), 3238–3260 (2016).

47. McKenzie, D., Jackson, J. & Priestley, K. Thermal structure of oceanic and continental lithosphere. *Earth Planet. Sci. Lett.* **233**(3), 337–349 (2005).

48. Bouhifd, M. A., Andrault, D., Fiquet, G. & Richet, P. Thermal expansion of forsterite up to the melting point. *Geophys. Res. Lett.* **23**(10), 1143–1146 (1996).

49. Hacker, B. R. & Abers, G. A. Subduction Factory 3: An Excel worksheet and macro for calculating the densities, seismic wave speeds, and H_2_O contents of minerals and rocks at pressure and temperature. *Geochem. Geophys. Geosyst.* **5**(1), Q01005 (2004).

50. Berman, R. G. & Aranovich, L. Y. Optimized standard state and solution properties of minerals. *Contrib. Mineral. Petrol.* **126**(1–2), 1–24 (1996).

51. Dziewonski, A. M. & Anderson, D. L. Preliminary reference Earth model. *Phis. Earth Planet. Int.* **25**(4), 297–356 (1981).

52. Omuralieva, A. M., Hasegawa, A., Matsuzawa, T., Nakajima, J. & Okada, T. Lateral variation of the cutoff depth of shallow earthquakes beneath the Japan Islands and its implications for seismogenesis. *Tectonophysics* **518–521**, 93–105 (2012).

53. Tanaka, A., Yamano, M., Yano, Y. & Sasada, M. Geothermal gradient and heat flow data in and around Japan (I): Appraisal of heat flow from geothermal gradient data. *Earth Planets Space* **56**(12), 1191–1194 (2004).

54. Nishimoto, S., Ishikawa, M., Arima, M., Yoshida, T. & Nakajima, J. Simultaneous high P-T measurements of ultrasonic compressional and shear wave velocities in Ichino-megata mafic xenoliths: Their bearings on seismic velocity perturbations in lower crust of northeast Japan arc. *J. Geophys. Res.* **113**(B12), B12212 (2008).

55. Boettcher, A. L. & Wyllie, P. J. Melting of granite with excess water to 30 kilobars pressure. *J. Geol.* **76**(2), 235–244 (1968).

56. Yoshioka, S. & Sanshadokoro, H. Numerical simulations of deformation and dynamics of horizontally lying slabs. *Geophys. J. Int.* **151**(1), 69–82 (2002).

57. Hirth, G. & Kohlstedt, D. L. Water in the oceanic upper mantle: implications for rheology, melt extraction and the evolution of the lithosphere. *Earth Planet. Sci. Lett.* **144**(1), 93–108 (1996).

58. Karato, S. On the origin of the asthenosphere. *Earth Planet. Sci. Lett.* **321–322**, 95–103 (2012).

59. Kohlstedt, D. L., Keppler, H. & Rubie, D. C. Solubility of water in the *α*, *β* and *γ* phases of (Mg,Fe)_2_SiO_4_. *Contrib. Mineral. Petrol.* **123**(4), 345–357 (1996).

60. Iwamori, H. Transportation of H_2_O beneath the Japan arcs and its implications for global water circulation. *Chem. Geol.* **239**(3–4), 182–198 (2007).

61. Abers, G. A., van Keken, P. E. & Hacker, B. R. The cold and relatively dry nature of mantle forearcs in subduction zones. *Nat. Geosci.* **10**, 333–337 (2017).

62. Hacker, B. R., Peacock, S. M., Abers, G. A. & Holloway, S. D. Subduction factory 2. Are intermediate-depth earthquakes in subducting slabs linked to metamorphic dehydration reactions? *J. Geophys. Res.* **108**(B1), 2030 (2003).

63. Anderson, D. L. *Theory of the Earth*, (Blackwell Scientific Publications, Oxford, 1989).

64. Čížková, H., van den Berg, A. P., Spakman, W. & Matyska, C. The viscosity of Earth’s lower mantle inferred from sinking speed of subducted lithosphere. *Phis. Earth Planet. Int.* **200–201**, 56–62 (2012).

65. Rosenberg, C. L., Medvedev, S. & Handy, M. R. Effects of melting on faulting and continental deformation. In: Handy, M. R., Hirth, G. & Hovius, N. (Eds.), *Tectonic Faults: Agents of Change on a Dynamic Earth., Dahlem Workshop Rep.* **95** (The MIT Press, Cambridge, Mass., 2007).

66. Kohlstedt, D. L. Partial melting and deformation. In: Karato, S. & Wenk, H.-R. (Eds.), *Plastic Deformation of Minerals and Rocks., Rev. Mineral. Geochem.* **51** (Mineralogical Society of America, Washington DC, 2002).

67. Wessel, P. & Smith, W. H. F. New, improved version of generic mapping tools released. *Eos, Trans. Am. Geophys. Union* **79**(47), 579–579 (1998).
